# Social inequalities and hospital admission for unintentional injury in young children in Scotland: A nationwide linked cohort study

**DOI:** 10.1016/j.lanepe.2021.100117

**Published:** 2021-07

**Authors:** Paul M. Henery, Ruth Dundas, S. Vittal Katikireddi, Alastair Leyland, Rachael Wood, Anna Pearce

**Affiliations:** a MRC/CSO Social and Public Health Sciences Unit, University of Glasgow, United Kingdom; b NHS National Services Scotland, Public Health Scotland, United Kingdom; c Centre for Clinical Brain Sciences, University of Edinburgh, United Kingdom

## Abstract

**Background:**

Unintentional injury is a leading cause of death/disability, with more disadvantaged children at greater risk. Understanding how inequalities vary by injury type, age, severity, and place of injury, can inform prevention.

**Methods:**

For all Scotland-born children 2009-2013 (n=195,184), hospital admissions for unintentional injury (HAUI) were linked to socioeconomic circumstances (SECs) at birth: area deprivation via the Scottish Index of Multiple Deprivation (SIMD), mother's occupational social class, parents’ relationship status. HAUI was examined from birth-five, and during infancy. We examined HAUI frequency, severity, injury type, and injury location (home vs. elsewhere). We estimated relative inequalities using the relative indices of inequality (RII, 95% CIs), before and after adjusting for demographics and other non-mediating SECs.

**Findings:**

More disadvantaged children were at greater risk of any HAUI from birth-five, RII: 1•59(1•49-1•70), 1•74(1•62-1•86), 1•97(1•84-2•12) for area deprivation, maternal occupational social class, and relationship status respectively. These attenuated after adjustment (1•15 [1•06-1•24], 1.22 [1•12-1•33], 1.32 [1•21-1•44]). Inequalities were greater for severe (vs. non-severe), multiple (vs. one-off) and home (vs. other location) injuries. Similar patterns were seen in infancy, excluding SIMD-inequalities in falls, where infants living in more disadvantaged neighbourhoods were at lower risk (0•79 [0•62-1•00]). After adjustment, reverse SIMD-gradients were also observed for all injuries and poisonings.

**Interpretation:**

Children living in more disadvantaged households are more likely to be injured across multiple dimensions of HAUI in Scotland. Upstream interventions which tackle family-level disadvantage may be most effective at reducing childhood HAUI.

**Funding:**

Wellcome Trust, Medical Research Council, Scottish Government Chief Scientist Office.

**Research in context**

**Evidence before this study**

In March 2020, we searched PubMed and Scopus using search strings pertaining to socio-economic circumstances, children and unintentional injury (UI) with no time limits. The majority of studies were from Europe (in particular, the United Kingdom). We found evidence of inequalities in childhood UI according to various socio-economic circumstances (SECs), for different injury types and injury locations (e.g. home). While existing research is collectively wide ranging, it is fragmented, with a lack of research examining multiple aspects of SECs, injury types, injury location, severity and frequency in one population. This is needed to understand where the largest inequalities lie and thus where policy efforts should focus. Additionally, there was a lack of research examining inequalities in unintentional injury in infancy, despite death rates from injury being highest in this group.

**Added value of this study**

To our knowledge, this is the first population-based study which directly compares inequalities in hospital admissions for unintentional injury (HAUI) in infancy and preschool age, for multiple SECs, injury types, and injury locations and according to differing levels of severity and frequency using hospitalisation data. We present relative and absolute inequalities between the most and least advantaged groups in infancy, from age one to five years, and across the whole period (birth to five years). Of 9,666 children with HAUI by the age of five, 5% experienced more than one injury and 5% had a severe injury (requiring transfer to another hospital/ward, rather than discharge to home, or resulting in death). Large relative inequalities in childhood were almost universally found regardless of severity, frequency, injury location, and injury type, for all SEC measures examined. Inequalities tended to be greater for severe and multiple injuries (compared to non-severe and one-off injuries) and for injuries occurring to children in the home (vs. elsewhere). Similar patterns were seen in infancy with one exception: a reverse socio-economic gradient in falls was observed according to area disadvantage, with those living in the least disadvantaged neighbourhoods at greater risk of falls. A similar reverse social gradient (according to area deprivation) emerged for all injuries and poisonings after adjustment for covariates, occupational social class and relationship status.

**Implications of all the available evidence**

Despite successful interventions to reduce the incidence of HAUI in Scotland, the UK and across the European region, children experiencing socioeconomic disadvantage remain at higher risk and especially so for severe, frequent and home-based injuries. In the current study we found evidence that infants living in disadvantaged areas were at a reduced risk of certain injury types, although in some instances this was only after adjustment for confounding and household SECs. There are very limited published data on socioeconomic inequalities in UI among infants, and so replication of this finding is needed. These results, in their entirety, indicate that while targeting disadvantaged areas (and households) with interventions known to prevent UIs may reduce inequalities (as this is where HAUI tends to be more prevalent), upstream policies, including those to alleviate household-level socio-economic disadvantage in young families, will likely be most effective. Such efforts will also benefit a multitude of other outcomes.

Alt-text: Unlabelled box

## 1 Introduction

The World Health Organisation (WHO) recognises unintentional injury (UI) as a major public health issue [1]. UI in childhood can cause disability, leading to lifelong impact on the child and their families, and was responsible for over half a million childhood deaths worldwide in 2016 [2]. Despite being largely preventable, the scale of UIs in childhood persists, though the death rate in high-income countries has decreased in recent years [3]. Children from more disadvantaged backgrounds are more likely to experience and die from UI. This is a universal finding across Europe and other high income countries, regardless of age range, severity or type of injury under study [4,5,6,7,8,9,10,11,12]. In general, health inequalities are thought to arise through material, psychosocial and behavioural-cultural pathways, as well as the wider physical, political and economic environment [4]. The Haddon Matrix, which attributes the causes of unintentional injury to the host, agent or environment, can shed light on why we see inequalities in UI specifically. For example, Campbell et al found that the child's physical environment (e.g. safety of the home, childcare and neighbourhood environments), factors that may affect supervision (e.g. maternal mental health, caregiver alcohol consumption, social support networks) and the child's abilities and behaviours (e.g. hyperactivity, hearing and sight) all contribute to inequalities in childhood UI. Inequalities in UI are seen across a range of socio-economic measures, including parental education, income [4], family structure [11], and neighbourhood deprivation [5,10]. None of these measures can encapsulate the complexity of families’ socio-economic circumstances in their entirety, and they will have commonalities (for example lone parent families and those living in deprived neighbourhoods will have, on average, lower incomes and home ownership rates than couple families and those living in advantaged neighbourhoods). Nevertheless, it is helpful to consider whether inequalities persist across different measures, since each may reflect different elements of socio-economic disadvantage and potentially point towards different mechanisms through which inequality may arise. For example, areas of high deprivation will have poorer physical environments and local services which could influence injury, such as traffic density, accessibility to high quality childcare, and availability of safe play areas. On the other hand, socio-economic characteristics of parents and the household may have a more direct influence on the quality of the home environment and caregiver attributes that can affect parenting capacity, such as mental distress and lower physical health. A review by Laflamme et al indicated that the vast majority of studies investigating inequalities in specific types of UI only examined one aspect of socio-economic inequality (with most relying on area-level measures) [7].

Although infants are less likely to be unintentionally injured than older children, mortality rates are considerably higher. In 2004, infants were three times as likely to die of an UI than 5-9 year olds in Europe [13]. Despite this, very few studies have examined inequalities in UI specifically among infants, with the majority including them with other ages [6,9] or excluding infants entirely [4,14]. It is important to differentiate social inequalities in injuries at different ages in childhood, as children's experience of their home and neighbourhood environments change as they develop and as their physical independence increases [15]; material mechanisms (such as via lower resources at home) could lead to greater risks for young children, and environmental factors (such as residence in areas of high traffic) could put older children in greater danger.

Most research has focused on experience of any injury regardless of type [4,16,17,7], with studies examining specific injury types biased towards road traffic accidents, burns and falls. There is some evidence that inequalities vary by injury type, with wider inequalities in transport-related accidents [7,9] burns [6,9,10], cuts, or poisonings [9] compared to other injury types such as falls or drowning [9]. However as mentioned above, most do not unilaterally include infants or do not differentiate infants from other ages [7].

Injury frequency and severity is less widely studied, although there is evidence that inequalities are wider for more severe injuries (in children aged under 14) [6] or frequent injuries (in children aged up to 5 years) [18]. Inequalities may also differ according to location where injury occurred (e.g. at home, in cars/on roads). A home environment can present hazards to small children and UIs in the home are leading causes of death in children aged under five. A study of UI hospitalisations occurring in Quebec, 2002-04 to children aged 0-14, indicated that inequalities in home UI are higher than those occurring in sports and recreational facilities and public buildings [6].This requires replication in other countries and in more recent samples (particularly due to the dramatic increase in centre-based childcare that has occurred since the early 2000s) [19].

Inequalities in UI are universally observed but may vary by age, the element of socio-economic circumstances (SECs) examined, and injury type, severity, frequency and location. However, few studies have examined how inequalities differ across these parameters within the same dataset or population. This will lead to a better understanding of the population burden of inequalities in UI, which can inform policy content and targeting across the European region. The current study aims to address this gap, examining hospital admission due to UI (HAUI) in a Scotland-wide administrative cohort.

The primary aim of the study was as to ascertain whether inequalities in hospital admission for unintentional injury in childhood were observed across multiple indicators of socio-economic circumstances. Our secondary aims were to determine whether inequalities in hospital admission for unintentional injury persisted across a) age group, b) type of injury, c) severity and frequency of injury, and d) location of injury.

## 2 Methods

### 2.1 Data sources

Analyses were carried out in an administrative cohort of all children born in Scotland, September 2009-March 2013 (n=202,757) for whom an individual identifier was available (the Community Health Index (CHI), which is assigned to all NHS patients in Scotland (approx. ~99% population)). The cohort was created by linking several administrative datasets. Socio-economic information was obtained from National Records of Scotland (NRS) vital event (birth) records. Demographic and birth characteristics were available from hospital maternity records (Scottish Morbidity Record 02 (SMR02)) or home births (Scottish Birth Record (SBR)). The Scottish Morbidity Record 01 (SMR01) captures hospital admissions from UI up until age five. We could not censor for death or emigration as we did not have access to this information; as a result, a small number of cases may have been classified as having not been hospitalised for an injury when they were in fact ineligible. The data were provided, with unique IDs derived from the CHI, by Information Services Division (ISD) Scotland following Public Benefit and Privacy Panel for Health (PBPP) approval (1617-0152); no ethical clearance was required.

### 2.2 Measures

#### 2.2.1 Outcomes: Hospital admission for unintentional injury (HAUI)

The SMR01, used to generate our injury measure, is an administrative dataset which captures all inpatient admissions to non-obstetric and non-psychiatric hospitals in Scotland, with each episode of inpatient care comprising an individual record [20]. The SMR01 is generally coded accurately [21]; in particular, variables such as speciality and admission type have accuracy of >90%.

Our primary outcome measure is hospital admission from unintentional injury (HAUI), i.e. injury as a result of an accident, as opposed to child maltreatment or intentional harm. These were identified with SMR admission code 1E “Accidental Injury”. Less severe injuries are not captured, such as those that were managed in the home, that were dealt with in primary care, or that were assessed at the emergency department to not require admission.

The following HAUI outcomes were examined over the entire period (birth-five years) and divided into infancy and one-five years.

• *Any injury*: child admitted to hospital for one or more unintentional injuries (yes, no).
• *Multiple injury:* number of admissions recorded, categorised as 0-1 or 2+ admissions.
• *Severe injury:* discharge destination was used as a proxy for unintentional injury severity. Discharge to a home setting was classified as non-severe; transfer to another hospital or ward (rather than to home) was classified as severe. The baseline was no or non-severe injury.
• *Injury type*: the following unintentional injury types, based on ICD codes (Appendix A2) were examined as independent binary measures (since children could experience more than one injury type): falls, strikes/cuts/piercings/crushes (SCC – as combined in the raw data), scalds, and poisonings. Transport-related injuries, drowning/submersion, threats to breathing, and smoke/fire/flame-related injuries were infrequent (<0•01% were admitted for these injuries) and so were not examined.
• *Injury location:* two independent binary measures were constructed –whether the child had experienced an unintentional injury which took place in the child's home location and whether the child had experienced an unintentional injury which took place in non-home locations such as a childminder's home, at school or nursery (collapsed from road traffic, work and other locations due to low frequency).

#### 2.2.2 Exposure: Socio-economic circumstance (SECs)

There is no one measure which can capture the complexity of family SECs in childhood [22]. Therefore three measures were examined, all derived from the NRS birth registration dataset at the time of the child's birth and all potentially capturing slightly different elements of SECs.

The 2012 version of the Scottish Index of Multiple Deprivation (SIMD), based on the “data zone”, a Scottish measure of small neighbourhood sub-units, and derived from full residential postcode, was used as a relative measure of area deprivation. Quintiles were used for descriptive analyses and deciles used in regression analyses. The SIMD is a widely used measure, describing social characteristics of neighbourhoods; crime levels, housing, income levels and employment rates indices for these domains are also available in addition to the multiple deprivation measure [23].

The National Statistics Socio-economic Classification (NS-SEC) represents the occupational social class of the mother: never worked/unemployed/student/other, routine/manual, intermediate, higher managerial/administrative/professional. It is a commonly used proxy for family socio-economic circumstances, as it captures employment relations and work conditions, and can be related to other characteristics such as income [24]. The father's NS-SEC was not used because it is not reported for sole birth registrations, who are more likely to be highly disadvantaged. However, as a sensitivity analysis we created a measure which took on the highest occupational social class of the mother or father, where both were registered (or for sole birth registrations, the occupational social class of the lone parent). The pattern of results was extremely similar to those for mother's occupational social class and so are not reported.

Birth registration details were used to capture the relationship status of the parents. This measure distinguishes between a number of family structures: married, cohabiting (joint registration, unmarried), separated (joint registration, living apart) and sole registration (only one parent registered the birth). Family structure is a strong indicator of poverty [25]. Descriptive analyses (Appendix A1) confirmed that relationship status could be used as an ordinal variable.

### 2.3 Covariates

We adjusted for variables which affect HAUI and are unlikely to mediate the association between current SECs and injuries– child sex, singleton or multiple births, and number of older siblings (interval). Derived from the SMR02 and SBR, these variables are related to neither the exposure or the outcome and do not lie on the causal pathway. We also adjusted for two potential common causes of SEC and UI, i.e. confounders [26], from NRS births. Mother's age at first live birth in years (interval, derived from date data for all births for mothers of the cohort children that occurred since 1980) and country of birth (Scotland, rest of UK & Ireland, other – due to low cell counts). Data were only available for births taking place in Scotland, therefore the mother's age at first live birth and older siblings variables did not take into account births outside the country.

**Fig. 1:**
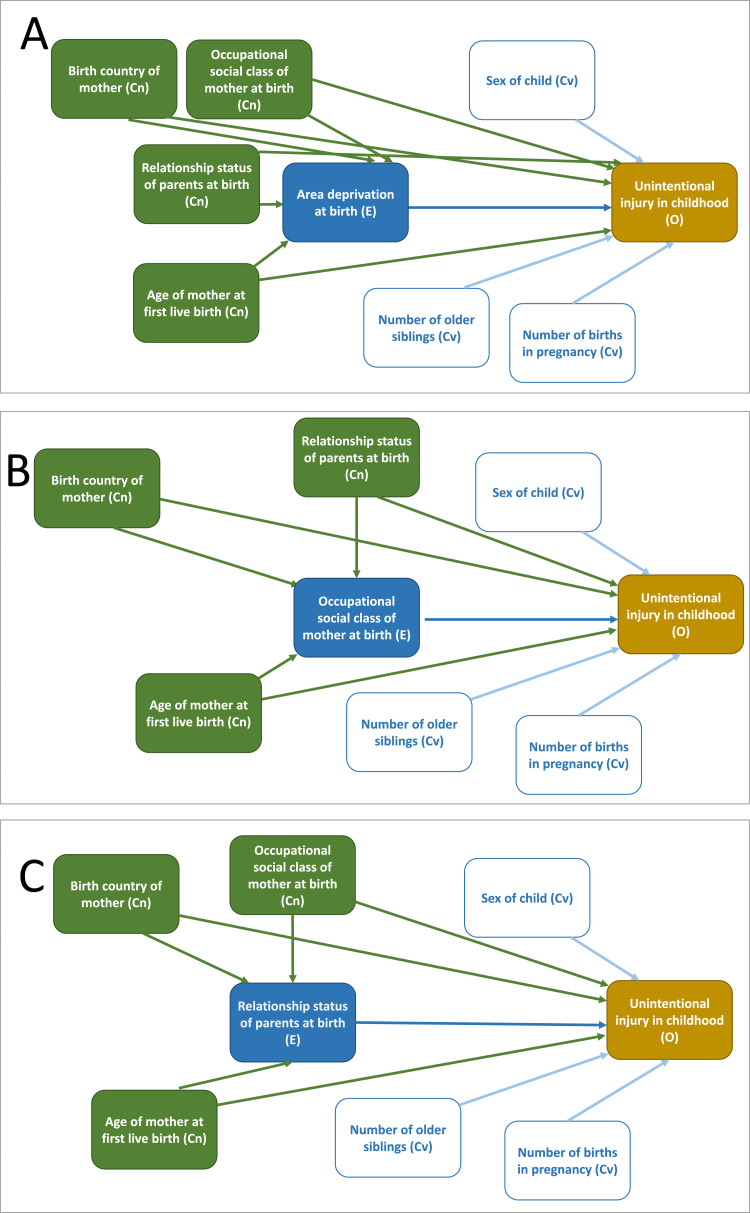
Directed acyclic graphs for the proposed causal relationship between socio-economic circumstances* and hospital admission for unintentional injury * Graph A: Area deprivation, Graph B: occupational social class, Graph C: relationship status of parents ** Because the direction between occupational social class and relationship status is uncertain, a conservative approach was taken, assuming that relationship status is a cause of occupational social class in B and that occupational social class is a cause of relationship status in C.

The hypothesised relationships between the variables included in the analysis are shown in the Directed Acyclic Graphs (Fig. 1). Age of the mother at first live birth and birth country of mother have been classified as confounders of all three SECs, but we acknowledge that it is possible that these factors may have been, for some families, a consequence rather than a cause of SECs. We therefore draw the readers’ attention to both the unadjusted and adjusted analyses. Further detail of the stages of adjustment and their interpretation are given in Statistical Analysis.

### 2.4 Statistical analyses

Descriptive analyses consisted of univariate frequency distributions, means, or medians as appropriate (Appendix A3), as well as bivariate analyses between all exposures, outcomes, and covariates (Appendix A1). Inequalities in all HAUI outcomes were assessed using relative (RII) indices of inequality (and 95% confidence intervals (CIs)), according to the three SEC measures. The RII is the ratio of prevalence of HAUI between the notionally most and least advantaged. The RII uses information from each category and not just the extremes of the distribution [27] and enable comparisons between different SEC measures. We also estimated absolute inequalities using the slope index of inequality (SII); since the patterns of absolute inequalities were very similar to those for relative inequality these are reported in the appendices (Appendix A4). The default regression-based confidence intervals for RII and SII may be underestimates [28]; however, the differences are marginal.

RIIs and SIIs were estimated in Stata using probabilities derived using the “binreg” command (RII) or the post-estimation “adjrr” package following logistic (SII) regression models. First we estimated unadjusted models which identify which socio-economic groups are most likely to experience HAUI. This is of importance from a public health perspective and can be used to direct resources. Next, we estimated adjusted models, including 1) covariates, 2) covariates and other SEC measures that did not lie on the causal pathway. These adjusted models may shed some light on what aspects of social disadvantage may be contributing to inequalities HAUI, although we would advise caution interpreting these estimates causally, because disentangling the pathways between different socio-economic measures is complex. For example, we did not adjust for area deprivation when estimating inequalities in occupational social class or relationship status, because area deprivation may be a mediator of these relationships. Relationship status and occupational social class are considered to be confounders of area deprivation and mutual confounders of each other (as we believe the relative causality of each measure on the other to be roughly equal). See DAGs (Fig. 1). However, we acknowledge that area deprivation may also affect occupational social class and relationship status as well as the other way around. In this situation the adjusted analyses may underestimate inequality and we therefore provide results at different stages of adjustment for comparison. Bivariate relationships between HAUI covariates are shown in Appendix A5. When relevant, we ran tests to check for multicollinearity for model variables, using the variance inflation factor (VIF) and Condition Index.

**Fig. 2:**
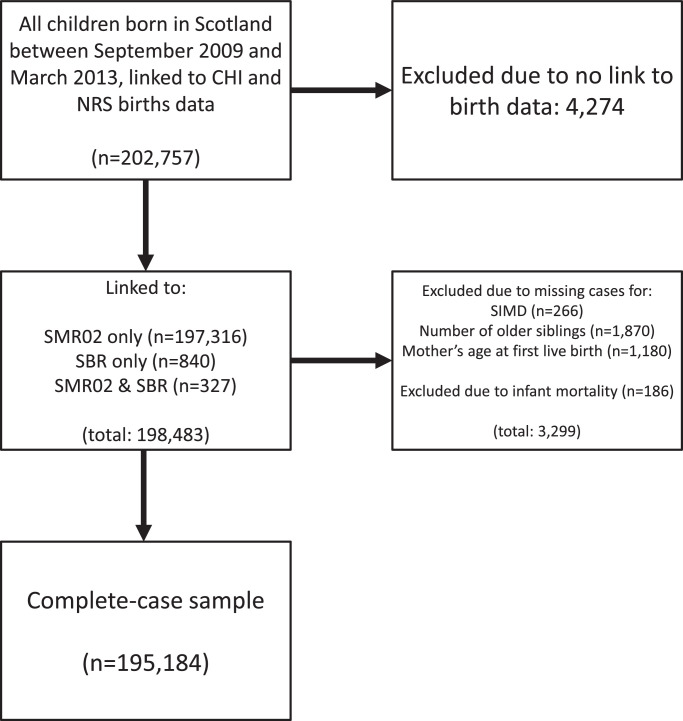
Flow diagram showing creation of analytic sample. CHI: Community Health Index; NRS: National Records of Scotland; SMR02: Scottish Morbidity Record 02; SBR: Scottish Birth Record; SIMD: Scottish Index of Multiple Deprivation.

We analysed a sample restricted to children who survived the first year of life with complete information for all variables (n=195,184, 96% original cohort). See Fig. 2 for further detail. The characteristics of the full and complete case sample were similar (Appendix A3). Analyses were carried out in Stata version 14.

**Fig. 3:**
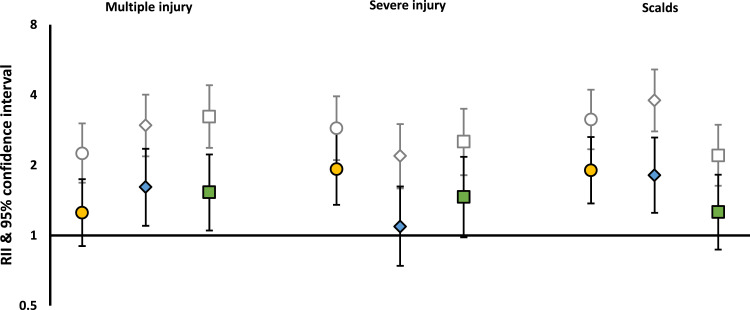
Unadjusted and adjusted* RIIs of hospital admission for unintentional injury from birth to age five, for injury types that could not be reported at specific ages due to statistical disclosure *Adjusted for sex of child, number of births in pregnancy, country of birth of mother, age of mother at first live birth, number of older siblings, SEC exposures (area deprivation was adjusted for occupational social class and relationship status; relationship status was adjusted for occupational social class; occupational social class was adjusted for relationship status).

### 2.5 Sensitivity analyses

We conducted a series of sensitivity analyses. Adjustment for health board (one of fourteen Scottish regions in which healthcare services are semi-autonomously delivered) in models which already adjusted for all other factors were carried out (Appendix A6) as we wished to examine whether potential differences in health services between areas might create spurious inequalities (especially by area deprivation) – however this made little difference to the overall results. We also examined inequalities in multiple (2+) injuries when compared to only those with one injury (Appendix A7) (excluding those who had not been injured at all, *n*= 9,512). When building fully adjusted models, we examined the impact of adjustment for each covariate in turn. Given an unexpected relationship between area deprivation and HAUI in infants, we present these separately adjusted models in Appendix A9. In Appendix A10 we consider whether fitting a linear association between SECs and HAUI in infants (the approach used to calculate the indices of inequality) was appropriate. Finally, to determine whether there were any impacts of clustering of children within families we considered whether children with another sibling in the dataset (~20%) were more likely to be admitted for unintentional injury, which they were but not at a level to disproportionately skew the results (data not shown).

### 2.6 Role of the funding source

The funder of the study had no role in study design, data collection, data analysis, data interpretation, or writing of the report. Henery, Pearce and Leyland had full access to the data and all authors had responsibility for submission to publication.

## 3 Results

### 3.1 Injury prevalence

Of the 195,184 children analysed, just under 5% (n=9,666) were hospitalised for at least one UI before age five years; children who were excluded from the complete case analysis were slightly less likely than the complete-case sample to have experienced an HAUI (Table 1) (NB characteristics for the full sample (complete case + excluded) are shown in Appendix A8). Of those, 16% had been injured by age one (<1% of the total sample). Of those who had been injured during the study period, 5% experienced one or more additional injuries and 5% had experienced a severe injury (making up 0•29% and 0•25% of the total sample respectively). Falls were the most common injury type, making up 45% of all injuries (with 2% of children being admitted to hospital for a fall-specific HAUI). Children were more likely to have an injury at home (3•2%) compared to non-home locations (1•9%), with similar patterns among infants (0•6% and 0•2%).

**Table 1:** Frequency distributions for injuries at from birth to age five, infancy and age one to five

Injury	Complete case sample (n=195,184)						Excluded sample (n=7,573)					
	Birth to age 5		Infancy only		Age 1 to 5 only		Birth to age 5		Infancy only		Age 1 to 5 only	
**Any injury**	9,666	4•95%	1,502	0•77%	8,267	4•24%	344	4.54%	49	0.65%	298	3.94%
**Multiple (2+) injuries**	560	0•29%	NA	NA	NA	NA	17	0.22%	NA	NA	NA	NA
**Severe injury**	497	0•25%	NA	NA	NA	NA	14	0.18%	NA	NA	NA	NA
**Transport-related**	147	0•08%	NA	NA	NA	NA	NA	NA	NA	NA	NA	NA
**Falls**	4,308	2•21%	829	0•42%	3,507	1•80%	137	1.81%	31	0.41%	106	1.40%
**Struck, cut, crush**	1,712	0•88%	206	0•11%	1,510	0•77%	74	0.98%	NA	NA	71	0.94%
**Drowning/submersion**	11	0•01%	NA	NA	NA	NA	NA	NA	NA	NA	NA	NA
**Threats to breathing**	58	0•03%	NA	NA	NA	NA	NA	NA	NA	NA	NA	NA
**Smoke/fire**	43	0•02%	NA	NA	NA	NA	NA	NA	NA	NA	NA	NA
**Scalds**	591	0•30%	NA	NA	NA	NA	20	0.26%	NA	NA	NA	NA
**Poisonings**	1,650	0•85%	100	0•05%	1,551	0•79%	63	0.83%	NA	NA	58	0.77%
**Admission from home**	6,318	3•24%	1,232	0•63%	5,141	2•63%	234	3.09%	38	0.50%	199	2.63%
**Admission from non-home location**	3,789	1•94%	383	0•20%	3,415	1•75%	126	1.66%	15	0.20%	111	1.47%

Cells containing “NA” have been suppressed either because they, or cells in the same analyses, have values <10, or because the authors elected not to run the analysis due to low prevalence

### 3.2 Socio-economic inequalities in any injury, multiple injuries and severe injuries

Children from more disadvantaged backgrounds were more likely to experience any HAUI between birth and age five (Table 2, Appendix A1). For example, relative inequalities for any HAUI were: 1•59 (1•49-1•70), 1•74 (1•62-1•86), 1•97 (1•84-2•12) for area deprivation, occupational social class and relationship status respectively (Table 2). These were attenuated after adjustment for covariates and other SECs (where relevant) indicating the multi-faceted nature of these inequalities (fully adjusted RIIs: 1•15 [1•06-1•24], 1•22 [1•12-1•33], 1•32 [1•21-1•44] for area deprivation, occupational social class and relationship status respectively). Further adjusting for area deprivation (which was not included in the main adjustment set as it was considered to be a potential confounder) in occupational social class and relationship status models reduced the RIIs only marginally (1•19 [1•09-1•30], 1•29 [1•18-1•41] respectively (data not shown)). Inequalities were also seen for multiple and severe injuries (Table 2, Figure 3).

**Table 2:** Relative (RII) indices of inequality and 95% confidence intervals for the difference between least and most advantaged children according to each socio-economic circumstance (SEC) indicator for any (vs. none) multiple (vs. 0-1) and severe (vs. none/non-severe) injury, before and after adjustment

Injury outcome		Socio-economic circumstances	Unadjusted		Adjusted for covariates*		Adjusted for covariates* and relevant SECs⁎⁎	
			RII	95% C.I.	RII	95% C.I.	RII	95% C.I.
**Any injury**	**Birth to age 5**	**Area deprivation**	1•59	1•49 to 1•70	1•24	1•15 to 1•34	1•15	1•06 to 1•24
		**Social class**	1•74	1•62 to 1•86	1•30	1•20 to 1•42	1•22	1•12 to 1•33
		**Relationship status**	1•97	1•84 to 2•12	1•39	1•28 to 1•51	1•32	1•21 to 1•44
	**Infancy only**	**Area deprivation**	1•20	1•01 to 1•43	0•89	0•74 to 1•08	0•77	0•63 to 0•93
		**Social class**	1•84	1•53 to 2•21	1•36	1•09 to 1•69	1•19	0•95 to 1•49
		**Relationship status**	2•42	2•01 to 2•92	1•82	1•46 to 2•26	1•74	1•39 to 2•18
	**Age 1 to 5 only**	**Area deprivation**	1•70	1•57 to 1•83	1•33	1•22 to 1•44	1•24	1•14 to 1•35
		**Social class**	1•73	1•61 to 1•87	1•30	1•19 to 1•42	1•22	1•11 to 1•35
		**Relationship status**	1•94	1•79 to 2•10	1•34	1•23 to 1•47	1•28	1•16 to 1•41
**Multiple injuries**⁎⁎⁎	**Birth to age 5**	**Area deprivation**	2•25	1•68 to 3•02	1•44	1•05 to 1•98	1•25	0•90 to 1•74
		**Social class**	2•96	2•18 to 4•01	1•77	1•23 to 2•56	1•61	1•10 to 2•35
		**Relationship status**	3•23	2•37 to 4•40	1•70	1•18 to 2•45	1•53	1•05 to 2•22
**Severe injury**⁎⁎⁎	**Birth to age 5**	**Area deprivation**	2•88	2•10 to 3•95	2•01	1•43 to 2•82	1•92	1•35 to 2•74
		**Social class**	2•19	1•59 to 3•00	1•19	0•81 to 1•75	1•09	0•74 to 1•62
		**Relationship status**	2•52	1•81 to 3•49	1•49	1•01 to 2•19	1•46	0•98 to 2•17

⁎ Adjusted for sex of child, number of births in pregnancy, country of birth of mother, age of mother at first live birth, number of older siblings

⁎⁎ Adjusted for other relevant SEC exposures (area deprivation was adjusted for social class and relationship status only; relationship status was adjusted for social class; social class was adjusted for relationship status)

⁎⁎⁎ Analyses for infancy and early childhood were not run due to low case numbers

Household-level inequalities in infancy were similar to those seen from birth to age five (Table 2). That is, there were inequalities in all injury outcomes according to occupational and relationship status. However, whilst similar results were found for area deprivation in cross-tabulations (Appendix A1) and unadjusted models (RII:1•20 [1•01-1•43]), after adjustment for covariates and other SECs a reverse social gradient was found (fully adjusted RII 0•77 [0•63-0•93]). Adjustment for age of mother at first live birth was principally responsible for this change in direction, although adjustment for individual SEC variables (occupational social class and relationship status) also attenuated the associations to the null (Appendix A9). Reversal of effects such as this can be caused by multi-collinearity, however we examined the Condition Index and VIFs, finding no evidence of strong multicollinearity (multicollinearity is defined as a VIF of 5 or higher and a Condition Index of 10 or higher, with strong collinearity defined as a Condition Index of 30 or higher) [29]; we found a Condition Index of 18.76 and VIFs of 1.25, 1.53, 1.41 and 1.67 for SIMD, NS-SEC, relationship status and age of the mother respectively. This along with the consistent width of the CIs, suggests this effect could not be explained by multi-collinearity. Furthermore, while further investigation showed that the relationship between area deprivation and HAUI was not strictly linear (Appendix A10), children living in less advantaged areas remained less likely to be admitted to hospital than those living in the most advantaged quintile.

HAUI occurring between one-five years (Table 2) produced broadly similar results compared to birth-five.

### 3.3 Socio-economic inequalities in types of injury

Children from more disadvantaged backgrounds were more likely to be admitted to hospital for all injury types (falls, scalds, poisonings, striking/cutting/crushing injuries) between birth and age five, both before and after adjustment (Table 3). Inequalities in home locations tended to be higher than for non-home locations.

**Table 3:** Relative (RII) indices of inequality and confidence interval of difference between least and most advantaged children for each SEC indicator, for types of injury and geographical injury locations in unadjusted, adjusted for covariates* and adjusted for exposure⁎⁎ models, in all age groups

Injury outcome		SEC	Unadjusted		Adjusted for covariates*		Adjusted for covariates* and relevant SECs⁎⁎	
			RII	95% C.I.	RII	95% C.I.	RII	95% C.I.
**Falls**	**Birth to age 5**	**Area deprivation**	1•32	1•19 to 1•46	1•13	1•01 to 1•26	1•08	0•96 to 1•21
		**Social class**	1•29	1•16 to 1•44	1•10	0•97 to 1•25	1•03	0•90 to 1•17
		**Relationship status**	1•64	1•47 to 1•83	1•26	1•11 to 1•43	1•23	1•07 to 1•40
	**Infancy only**	**Area deprivation**	0•79	0•62 to 1•00	0•66	0•51 to 0•85	0•56	0•43 to 0•73
		**Social class**	1•41	1•11 to 1•80	1•31	0•98 to 1•76	1•30	0•96 to 1•77
		**Relationship status**	1•84	1•43 to 2•37	1•65	1•23 to 2•22	1•79	1•32 to 2•45
	**Age 1 to 5 only**	**Area deprivation**	1•50	1•34 to 1•68	1•29	1•14 to 1•46	1•26	1•11 to 1•44
		**Social class**	1•27	1•13 to 1•43	1•05	0•92 to 1•22	0•97	0•83 to 1•12
		**Relationship status**	1•62	1•44 to 1•83	1•20	1•04 to 1•38	1•14	0•98 to 1•32
**Striking, cutting, crushing injuries**	**Birth to age 5**	**Area deprivation**	1•66	1•41 to 1•96	1•27	1•06 to 1•52	1•14	0•95 to 1•38
		**Social class**	1•97	1•66 to 2•33	1•42	1•16 to 1•74	1•28	1•03 to 1•58
		**Relationship status**	2•05	1•72 to 2•44	1•51	1•23 to 1•85	1•37	1•11 to 1•70
	**Infancy only**	**Area deprivation**	1•93	1•19 to 3•12	1•21	0•71 to 2•04	1•00	0•58 to 1•72
		**Social class**	2•46	1•50 to 4•05	1•40	0•76 to 2•56	1•13	0•60 to 2•12
		**Relationship status**	4•74	2•83 to 7•94	2•52	1•37 to 4•63	2•45	1•30 to 4•63
	**Age 1 to 5 only**	**Area deprivation**	1•63	1•37 to 1•94	1•27	1•05 to 1•54	1•16	0•95 to 1•42
		**Social class**	1•91	1•59 to 2•29	1•42	1•14 to 1•76	1•30	1•03 to 1•63
		**Relationship status**	1•83	1•52 to 2•21	1•40	1•13 to 1•74	1•27	1•01 to 1•59
**Scalds**⁎⁎⁎	**Birth to age 5**	**Area deprivation**	3•09	2•32 to 4•14	2•19	1•60 to 2•98	1•88	1•36 to 2•59
		**Social class**	3•79	2•81 to 5•12	2•25	1•58 to 3•20	1•86	1•29 to 2•68
		**Relationship status**	2•20	1•63 to 2•97	1•69	1•19 to 2•40	1•27	0•88 to 1•82
**Poisonings**	**Birth to age 5**	**Area deprivation**	2•05	1•73 to 2•43	1•44	1•20 to 1•73	1•29	1•07 to 1•57
		**Social class**	2•18	1•83 to 2•59	1•46	1•18 to 1•81	1•27	1•02 to 1•59
		**Relationship status**	2•87	2•40 to 3•43	1•61	1•30 to 1•98	1•42	1•14 to 1•77
	**Infancy only**	**Area deprivation**	1•11	0•56 to 2•21	0•72	0•34 to 1•52	0•65	0•30 to 1•41
		**Social class**	1•68	0•83 to 3•41	1•01	0•43 to 2•37	0•96	0•39 to 2•35
		**Relationship status**	2•65	1•28 to 5•49	1•59	0•67 to 3•75	1•78	0•72 to 4•39
	**Age 1 to 5 only**	**Area deprivation**	2•14	1•80 to 2•55	1•51	1•25 to 1•83	1•36	1•11 to 1•65
		**Social class**	2•21	1•85 to 2•65	1•50	1•20 to 1•87	1•29	1•03 to 1•63
		**Relationship status**	2•89	2•40 to 3•48	1•61	1•29 to 2•00	1•40	1•11 to 1•76
**Home location**	**Birth to age 5**	**Area deprivation**	1•79	1•64 to 1•94	1•30	1•19 to 1•43	1•18	1•07 to 1•30
		**Social class**	2•07	1•90 to 2•26	1•42	1•28 to 1•58	1•28	1•15 to 1•43
		**Relationship status**	2•24	2•04 to 2•45	1•48	1•33 to 1•65	1•34	1•20 to 1•50
	**Infancy only**	**Area deprivation**	1•26	1•03 to 1•53	0•96	0•77 to 1•18	0•80	0•65 to 1•00
		**Social class**	1•93	1•58 to 2•36	1•46	1•15 to 1•86	1•31	1•02 to 1•69
		**Relationship status**	2•46	2•00 to 3•02	1•96	1•54 to 2•50	1•94	1•51 to 2•50
	**Age 1 to 5 only**	**Area deprivation**	1•96	1•79 to 2•16	1•41	1•27 to 1•57	1•30	1•17 to 1•45
		**Social class**	2•12	1•92 to 2•34	1•42	1•26 to 1•60	1•27	1•13 to 1•44
		**Relationship status**	2•21	2•00 to 2•44	1•39	1•24 to 1•57	1•23	1•09 to 1•39
**Non-home location**	**Birth to age 5**	**Area deprivation**	1•42	1•27 to 1•58	1•20	1•06 to 1•35	1•12	0•99 to 1•26
		**Social class**	1•45	1•30 to 1•63	1•23	1•07 to 1•41	1•13	0•98 to 1•31
		**Relationship status**	1•79	1•59 to 2•01	1•35	1•17 to 1•55	1•27	1•10 to 1•47
	**Infancy only**	**Area deprivation**	1•20	0•85 to 1•71	0•76	0•52 to 1•12	0•65	0•44 to 0•96
		**Social class**	2•37	1•65 to 3•42	1•58	1•01 to 2•45	1•52	0•96 to 2•41
		**Relationship status**	3•10	2•13 to 4•50	1•72	1•11 to 2•68	1•74	1•09 to 2•76
	**Age 1 to 5 only**	**Area deprivation**	1•45	1•29 to 1•62	1•26	1•11 to 1•42	1•19	1•04 to 1•35
		**Social class**	1•38	1•22 to 1•55	1•20	1•04 to 1•38	1•10	0•94 to 1•27
		**Relationship status**	1•69	1•49 to 1•91	1•31	1•13 to 1•51	1•23	1•05 to 1•43

⁎ Adjusted for sex of child, number of births in pregnancy, country of birth of mother, age of mother at first live birth, number of older siblings

⁎⁎ Adjusted for other relevant SEC exposures (area deprivation was adjusted for social class and relationship status only; relationship status was adjusted for social class; social class was adjusted for relationship status)

⁎⁎⁎ Analyses for infancy and early childhood were not run due to low case numbers

**Fig. 4:**
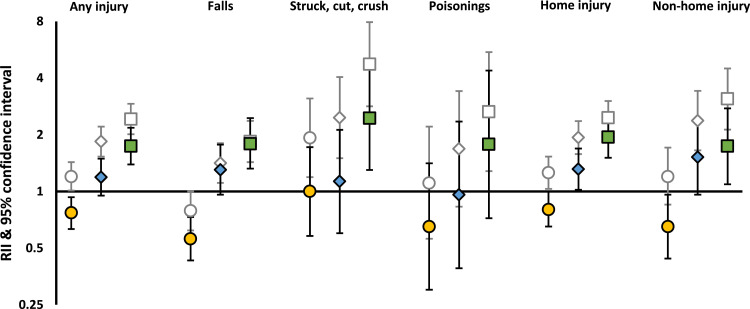
Unadjusted and adjusted* RIIs of hospital admission for unintentional injury all injuries, type of injury and physical location in infants *Adjusted for sex of child, number of births in pregnancy, country of birth of mother, age of mother at first live birth, number of older siblings, SEC exposures (area deprivation was adjusted for occupational social class and relationship status; relationship status was adjusted for occupational social class; occupational social class was adjusted for relationship status)

In infancy, household-level inequalities were observed for both occupational social class and relationship status (Fig. 4). Those living in deprived areas were also more likely to experience all types of HAUI before adjustment, with one exception: those living in more disadvantaged neighbourhoods were less likely to experience falls (RII: 0•79 [0•62-1•00]) and more so after adjustment (RII 0•56 [0•43-0•73]). A reduced risk for poisonings also emerged after adjustment, albeit it with very wide CIs (RII 0•65 [0•30-1•41]) (Fig. 4). After adjustment, children from the most disadvantaged areas were less likely to be admitted from non-home (RII 0•65 [0•44-0•96]) and home (RII 0•80 [0•65-1•00]) locations (Fig. 4).

**Fig. 5:**
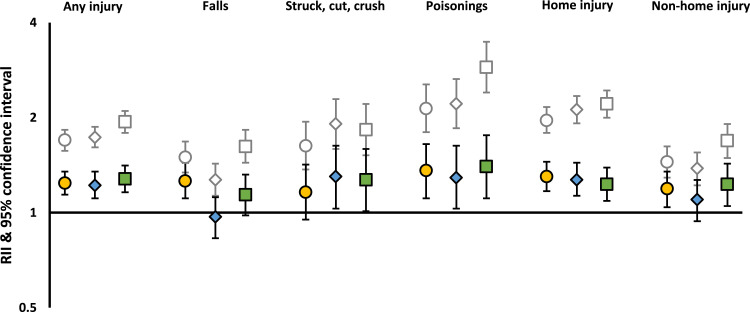
Unadjusted and adjusted* RIIs of hospital admission for unintentional injury from age one to five, by type of injury and SEC *Adjusted for sex of child, number of births in pregnancy, country of birth of mother, age of mother at first live birth, number of older siblings, SEC exposures (area deprivation was adjusted for occupational social class and relationship status; relationship status was adjusted for occupational social class; occupational social class was adjusted for relationship status)

Between age one and five, household-level inequalities were broadly similar to those seen from birth to age five (Fig. 5), and area deprivation was associated with injuries in the expected direction (those living in more disadvantaged areas at greater risk).

## 4 Discussion

Our analysis of a national administrative cohort of linked health data found large area- and household-level relative inequalities in HAUI between age one and five years regardless of severity, frequency, type, and location of injury. Disadvantaged children were more at risk of HAUI compared to advantaged children, and this persisted (though attenuated) after adjustment for confounders and other SECs (where relevant), indicating the multi-faceted nature of these inequalities.

Inequalities according to occupational social class and relationship status were consistently associated with HAUI among infants; those from disadvantaged backgrounds were more like to be admitted to hospital for all injuries, different injury types, such as falls and poisonings, and for injuries taking place both at home or elsewhere. A reverse socio-economic gradient according to neighbourhood deprivation was found for falls both before and after adjustment for covariates and other SECs, with similar findings emerging for all injuries and possibly poisonings after adjustment. This suggests that infants living in disadvantaged neighbourhoods were at lower risk of injury than those living in advantaged neighbourhoods after accounting for the fact that they tended to have younger mothers and live in less advantaged households.

Previous research has consistently demonstrated, across Europe and other high income countries, that inequalities exist in childhood UI, for any HAUI [4,7,30], specific injury types such as falls [7,17] or poisonings [7,9,10], in severe [6] and multiple [18] injuries, from home and other injury locations [6] and in both the preschool years [4,18] and infancy [16]. However, these have not been found within the same sample or population. Furthermore, the ages of children in each study varied considerably, with some looking at specific ages [4] and others collapsing infancy, toddlers and children into the same bracket [9, 17]. Inequalities in injuries requiring hospital admission that occurred at home tended to be greater than those occurring elsewhere, and this has been observed elsewhere [6]. Similarly, greater inequalities for multiple [18] and severe [6] injuries have also been reported in a very small number of studies. Previous research has found that inequalities persist across multiple measures of SECs, although with no consistent pattern for some aspects of SECs being more important than others [4, 8, 16,18]. We are cautious in directly comparing the size of inequality according to the measures we have examined, as they are subject to different sources and of degrees of measurement error. Nevertheless our findings indicate that all are important, thus demonstrating the pervasive and multi-faceted nature of inequalities in HAUI. It is likely that the different SEC measures capture different aspects of disadvantage to some degree, and therefore we might expect that the mechanisms through which they impact HAUI will also vary. Previous research, with data on a rich range of possible mechanisms, found that factors such as household safety equipment, maternal mental health, caregiver alcohol consumption and the child's socio-emotional wellbeing contributed to but could not entirely explain inequalities in UI [4]. Future research should seek to examine whether mechanisms vary across different SE measures, in order to gain a better understanding of how inequalities emerge and how they might be reduced.

To our knowledge, at the time of writing, no previous research has found a reverse socio-economic gradient in HAUI by area-level deprivation. For UI outcomes related to health service use, such as seeking primary health care or attendance at emergency departments, the inverse care law may lead to a reverse socio-economic gradient, potentially as a result of more socially advantaged parents being more aware of the importance of seeking medical attention when the child has suffered an injury, and pressing medical professions for an inpatient admission. For example, in the Millennium Cohort Study, mothers with higher academic qualifications were more likely to report an infant UI which required seeking the advice of a health professional, such as a GP [16]. This study also looked at neighbourhood deprivation, and while a reverse socio-economic gradient was not identified, those in the least deprived quintile were more likely to report an UI than those in the middle deciles of deprivation. However, we believe that the inverse care law would be less prominent for injuries that were sufficiently severe to require admission to hospital. Nevertheless it does remain possible that admission for UIs among infants, which may require more subjective assessments than for older children, may be more likely in well-resourced neighbourhoods. Future research should first seek to replicate this relationship, in the UK and elsewhere, before seeking to provide explanations for its existence.

We analysed linked data for almost all children born in Scotland 2009-2013, making it one of the largest studies to date. We were able to examine household-level as well as area-level SEC exposures (which is not ordinarily achievable using administrative data) and a range of outcomes including injury type, severity, and location. Importantly, we differentiated between injuries occurring in infancy and age one to five and in doing so identified some potentially complex and unexpected patterns. The finding that infants living in the most deprived neighbourhoods are at a lower risk of falls and possibly other injury types (after accounting for household-level SECs) may be spurious and should be replicated in other populations. As directed by our DAG, we adjusted for a range of baseline confounders, including mother's age at first live birth, assuming that age at first child influences relationship status, occupational social class and neighbourhood deprivation. It is possible that SECs could also affect age at first live birth, in which case the adjusted analyses will be over-adjusted. We adjusted for other SECs measures that were unlikely to be mediators, in that we assumed that relationship status and occupational social class would affect area deprivation rather than the other way around. Again this may represent an over-adjustment, but nevertheless helps to highlight the different socio-economic mechanisms through which injuries might be influenced.

In this paper we have examined hospital admissions for unintentional injury, which some might consider a limitation since it does not capture all injuries for which health care was not sought or for which admission to hospital was not required. As noted above, there is potential for differential admission rates following similar injuries; for example, parents of more advantaged children may be more likely to insist on admission, underestimating strength of inequality, or availability of health services may differ by geographical area. There is no one perfect measure of SEC; the three we have utilised in this paper all capture different aspects of SECs and all have limitations. Mother's occupational social class recorded at the birth of the cohort child may not reflect the highest occupation, if she has had children previously and this had affected her previous employment opportunities and decisions. That said, sensitivity analyses using the highest occupational social class of parents produced very similar results. The timepoint of SECs measurement (at the child's birth) may not reflect the circumstances at the time of injury occurred. For example, there is evidence that families from less advantaged families are more likely to move in the years following the birth of a child and that they are more likely to move to a nicer area [31]. If this is the case here, then we may have underestimated inequalities. A further limitation is that there may have been undocumented errors in the linkage process [32]. Previous studies investigating linkage errors in England [33] and Australia [34] with similar matching methods have found that preterm births and low birth weight children are less likely to be linked to mothers. Given that inequalities have been observed in both preterm and low birth weight children, our data may underestimate inequalities in HAUI by a very small amount.

It is possible that some children had left Scotland or died during the study period and thus we may have underestimated HAUI prevalence and inequalities (if these children were on average more likely to be injured). We used destination code (home vs. another ward / death) as a proxy for injury severity, which is an imperfect measure. For example, a child may have been transferred to another ward because of complications arising due to the existence of another condition. A very small number of children (4,274) were not linked to birth data and were therefore excluded from the cohort. However there was no consistent pattern in their SECs and so we believe this will have had limited impact on our findings. Our dataset only included children who were born in Scotland. Therefore injuries to children who were born outside Scotland but later moved there have not been captured. Furthermore, the mother's age at first live birth and the presence of older siblings will have been misrepresented for mothers who had recently moved to Scotland and had previous children in other countries. This may have affected the confounding impact of the age of mother and older siblings variables slightly, but this represents a very small proportion of the cohort. We also acknowledge that the selection of confounders is subjective and our DAGs may not accurately represent the true material, psychosocial, behavioural-cultural and environmental mechanisms underpinning SECs and HAUI. Adjustment for maternal age at first live birth and mutual adjustment for SECs in some models may have led to an underestimation of inequalities if they are mediators as opposed to confounders, hence our inclusion and reporting of the unadjusted results as well. Finally, it should be noted that whilst we have discussed causal mechanisms underpinning the impact of SECs on HAUI, we were unable to examine these with the data available.

Our unadjusted results indicated that disadvantaged households and disadvantaged neighbourhoods (in the majority of cases) had the highest rates of HAUI. Therefore, targeting interventions and policies known to prevent UI towards these groups could help to reduce inequalities. One-to-one parenting interventions, or home visitations have the potential to reduce likelihood of injury [35] – given that strong inequalities were found in home injury, these may be particularly effective. However it is widely accepted that upstream interventions hold the greatest potential for the reduction of health inequalities [36]. Unfortunately there is a general dearth of high quality research evaluating the impacts of upstream interventions from an inequalities perspective [36]. For childhood unintentional injuries, the evidence pertains mainly to road traffic collisions: legislation to enforce driving speed and drink drive limits, and bicycle helmet and seat-belt use (including child restraints), have reduced inequalities in road traffic injuries in children [13], as have traffic calming measures [37]. Public health guidelines [38], based on a review of interventions to reduce UI in childhood, recommend that planning takes account proximity of housing to fast traffic or a high incidence of on-street parking, which can present an increased risk of injury [39]. However, as we have seen in the current study, the large majority of unintentional injuries resulting in hospital admission from birth-five are now unrelated to traffic accidents in the UK. There are likely to be a myriad of other upstream changes that could thus reduce inequalities in unintentional injuries, including changes to schools, childcare centres and housing [40]. Research is lacking in this area, although evidence suggests that physical changes, such as use of safety equipment, to the school [41] or home environment [35] have the potential to reduce inequalities in UI. Inequalities in HAUI were found according to a range of SECs measures and different injury types, indicating that the mechanisms through which inequalities in injuries arise are numerous and complex; targeting specific mechanisms, such as home safety or high traffic in built up areas, may not be as effective as more wide-ranging interventions. This may be supported by our finding that neighbourhood level inequalities in infant HAUI were reversed after adjustment for household-level SECs, although we suggest that these first warrant replication in other data. Given that this study and other research across the continent identifies the multi-faceted mechanisms in which UI arises [7], we believe on balance that policies which focus primarily on reducing household-level social disadvantage among young families are likely to be most effective at reducing health inequalities [4,8], both in the UK and Europe more widely.

In conclusion, we found that large inequalities in childhood HAUI are pervasive in Scotland, persisting throughout the early years, across injury type, severity, frequency and location, and especially according to family-level SECs. Given the widespread nature of inequalities in UI, upstream efforts to reduce income inequality and improve housing environments may be most effective at reducing UI in the UK and across Europe.

## Contributions

PMH, AL and AP designed the study. PMH, AP and AL had full access to all the data in the study and take responsibility for the integrity of the data and the accuracy of the data analyses. PMH analysed the data. All authors interpreted the data. PMH drafted the manuscript. SVK, AL, RD, RW and AP critically revised the manuscript. AP obtained funding for the study.

## Data sharing

Data were only available to the authors in a safe setting due to being sensitive; however, syntax used to run analysis is available on request.

## Declaration of interests

The authors have no conflicts of interest to declare except for the funding noted in the funding statement.

## Appendix Supplementary materials

Image, application 1
