# Supplementary material for: Social inequalities and hospital admission for unintentional injury in young children in Scotland: A nationwide linked cohort study
Source: Lancet Reg Health Eur. 2021 May 10;6:100117. doi: 10.1016/j.lanepe.2021.100117 (PMC8278494; doi:10.1016/j.lanepe.2021.100117)
Supplement: Supplementary file 1 [file mmc1.docx]

**Social inequalities and hospital admission for unintentional injury in young children in Scotland: A nationwide linked cohort study (appendices)**

Appendix A1: Crosstabulations between all exposures and all injury outcomes, from birth to age five, in infancy and age one to five

| **Injury** | **Socio-economic circumstances** | **Category** | **All ages** | | **Infancy** | | **Early childhood** | |
| --- | --- | --- | --- | --- | --- | --- | --- | --- |
|  |  |  | **Freq.** | **Prop.** | **Freq.** | **Prop.** | **Freq.** | **Prop.** |
| **All injury** | **Area deprivation** | **1^st^ (most deprived)** | 3,031 | 5·92% | 437 | 0·85% | 2,641 | 5·16% |
|  |  | **2^nd^** | 2,083 | 5·04% | 316 | 0·77% | 1,789 | 4·33% |
|  |  | **3^rd^** | 1,752 | 4·67% | 268 | 0·71% | 1,498 | 4·00% |
|  |  | **4^th^** | 1,581 | 4·53% | 246 | 0·70% | 1,343 | 3·85% |
|  |  | **5^th^ (least deprived)** | 1,219 | 4·02% | 235 | 0·78% | 996 | 3·29% |
|  |  | **(p-value)** | <0·001 | | 0·084 | | <0·001 | |
|  | **Social class** | **Unemployed** | 2,553 | 6·09% | 427 | 1·02% | 2,160 | 5·15% |
|  |  | **Manual** | 2,796 | 5·34% | 409 | 0·78% | 2,422 | 4·62% |
|  |  | **Intermediate** | 1,843 | 4·59% | 275 | 0·69% | 1,580 | 3·94% |
|  |  | **Professional** | 2,474 | 4·07% | 391 | 0·64% | 2,105 | 3·47% |
|  |  | **(p-value)** | <0·001 | | <0·001 | | <0·001 | |
|  | **Relationship status** | **Sole registration** | 709 | 6·90% | 124 | 1·21% | 601 | 5·85% |
|  |  | **Joint reg, separated** | 1,511 | 6·48% | 235 | 1·01% | 1,297 | 5·57% |
|  |  | **Joint registration** | 3,516 | 5·30% | 575 | 0·87% | 2,983 | 4·49% |
|  |  | **Married** | 3,930 | 4·13% | 568 | 0·60% | 3,386 | 3·56% |
|  |  | **(p-value)** | <0·001 | | <0·001 | | <0·001 | |
| **Severe injury** | **Area deprivation** | **1st** | 181 | 0·35% | NA | NA | NA | NA |
|  |  | **2nd** | 112 | 0·27% | NA | NA | NA | NA |
|  |  | **3rd** | 95 | 0·25% | NA | NA | NA | NA |
|  |  | **4th** | 70 | 0·20% | NA | NA | NA | NA |
|  |  | **5th** | 39 | 0·13% | NA | NA | NA | NA |
|  |  | **(p-value)** | <0·001 | | NA | | NA | |
|  | **Social class** | **Unemployed** | 139 | 0·33% | NA | NA | NA | NA |
|  |  | **Manual** | 154 | 0·29% | NA | NA | NA | NA |
|  |  | **Intermediate** | 87 | 0·22% | NA | NA | NA | NA |
|  |  | **Professional** | 117 | 0·19% | NA | NA | NA | NA |
|  |  | **(p-value)** | <0·001 | | NA | | NA | |
|  | **Relationship status** | **Sole registration** | 47 | 0·46% | NA | NA | NA | NA |
|  |  | **Joint reg, separated** | 83 | 0·36% | NA | NA | NA | NA |
|  |  | **Joint registration** | 175 | 0·26% | NA | NA | NA | NA |
|  |  | **Married** | 192 | 0·20% | NA | NA | NA | NA |
|  |  | **(p-value)** | <0·001 | | NA | | NA | |
| **Multiple injury** | **Area deprivation** | **1^st^ (most deprived)** | 185 | 0·36% | NA | NA | NA | NA |
|  |  | **2^nd^** | 133 | 0·32% | NA | NA | NA | NA |
|  |  | **3^rd^** | 109 | 0·29% | NA | NA | NA | NA |
|  |  | **4^th^** | 85 | 0·24% | NA | NA | NA | NA |
|  |  | **5^th^ (least deprived)** | 48 | 0·16% | NA | NA | NA | NA |
|  |  | **(p-value)** | <0·001 | | NA | | NA | |
|  | **Social class** | **Unemployed** | 177 | 0·42% | NA | NA | NA | NA |
|  |  | **Manual** | 170 | 0·32% | NA | NA | NA | NA |
|  |  | **Intermediate** | 96 | 0·24% | NA | NA | NA | NA |
|  |  | **Professional** | 117 | 0·19% | NA | NA | NA | NA |
|  |  | **(p-value)** | <0·001 | | NA | | NA | |
|  | **Relationship status** | **Sole registration** | 54 | 0·53% | NA | NA | NA | NA |
|  |  | **Joint reg, separated** | 92 | 0·39% | NA | NA | NA | NA |
|  |  | **Joint registration** | 222 | 0·33% | NA | NA | NA | NA |
|  |  | **Married** | 192 | 0·20% | NA | NA | NA | NA |
|  |  | **(p-value)** | <0·001 | | NA | | NA | |
| **Transport** | **Area deprivation** | **1^st^ (most deprived)** | 49 | 0·10% | NA | NA | NA | NA |
|  |  | **2^nd^** | 39 | 0·09% | NA | NA | NA | NA |
|  |  | **3^rd^** | 23 | 0·06% | NA | NA | NA | NA |
|  |  | **4^th^** | 23 | 0·07% | NA | NA | NA | NA |
|  |  | **5^th^ (least deprived)** | 13 | 0·04% | NA | NA | NA | NA |
|  |  | **(p-value)** | 0·033 | | NA | | NA | |
|  | **Social class** | **Unemployed** | 48 | 0·11% | NA | NA | NA | NA |
|  |  | **Manual** | 37 | 0·07% | NA | NA | NA | NA |
|  |  | **Intermediate** | 29 | 0·07% | NA | NA | NA | NA |
|  |  | **Professional** | 33 | 0·05% | NA | NA | NA | NA |
|  |  | **(p-value)** | 0·006 | | NA | | NA | |
|  | **Relationship status** | **Sole registration** | NA | NA | NA | NA | NA | NA |
|  |  | **Joint reg, separated** | NA | NA | NA | NA | NA | NA |
|  |  | **Joint registration** | NA | NA | NA | NA | NA | NA |
|  |  | **Married** | NA | NA | NA | NA | NA | NA |
|  |  | **(p-value)** | NA | | NA | | NA | |
| **Falls** | **Area deprivation** | **1^st^ (most deprived)** | 1,278 | 2·50% | 207 | 0·40% | 1,080 | 2·11% |
|  |  | **2^nd^** | 908 | 2·20% | 171 | 0·41% | 750 | 1·82% |
|  |  | **3^rd^** | 794 | 2·12% | 144 | 0·38% | 652 | 1·74% |
|  |  | **4^th^** | 725 | 2·08% | 151 | 0·43% | 576 | 1·65% |
|  |  | **5^th^ (least deprived)** | 603 | 1·99% | 156 | 0·51% | 449 | 1·48% |
|  |  | **(p-value)** | <0·001 | | 0·093 | | <0·001 | |
|  | **Social class** | **Unemployed** | 1,018 | 2·43% | 224 | 0·53% | 804 | 1·92% |
|  |  | **Manual** | 1,189 | 2·27% | 211 | 0·40% | 986 | 1·88% |
|  |  | **Intermediate** | 892 | 2·22% | 148 | 0·37% | 747 | 1·86% |
|  |  | **Professional** | 1,209 | 1·99% | 246 | 0·41% | 970 | 1·60% |
|  |  | **(p-value)** | <0·001 | | <0·001 | | <0·001 | |
|  | **Relationship status** | **Sole registration** | 270 | 2·63% | 57 | 0·56% | 217 | 2·11% |
|  |  | **Joint reg, separated** | 643 | 2·76% | 122 | 0·52% | 526 | 2·26% |
|  |  | **Joint registration** | 1,556 | 2·34% | 310 | 0·47% | 1,261 | 1·90% |
|  |  | **Married** | 1,839 | 1·93% | 340 | 0·36% | 1,503 | 1·58% |
|  |  | **(p-value)** | <0·001 | | <0·001 | | <0·001 | |
| **Striking, cutting, crushing injuries** | **Area deprivation** | **1^st^ (most deprived)** | 556 | 1·09% | 73 | 0·14% | 485 | 0·95% |
|  |  | **2^nd^** | 361 | 0·87% | 43 | 0·10% | 318 | 0·77% |
|  |  | **3^rd^** | 296 | 0·79% | 34 | 0·09% | 263 | 0·70% |
|  |  | **4^th^** | 278 | 0·80% | 29 | 0·08% | 250 | 0·72% |
|  |  | **5^th^ (least deprived)** | 221 | 0·73% | 27 | 0·09% | 194 | 0·64% |
|  |  | **(p-value)** | <0·001 | | 0·042 | | <0·001 | |
|  | **Social class** | **Unemployed** | 488 | 1·16% | 63 | 0·15% | 425 | 1·01% |
|  |  | **Manual** | 482 | 0·92% | 57 | 0·11% | 428 | 0·82% |
|  |  | **Intermediate** | 313 | 0·78% | 41 | 0·10% | 273 | 0·68% |
|  |  | **Professional** | 429 | 0·71% | 45 | 0·07% | 384 | 0·63% |
|  |  | **(p-value)** | <0·001 | | 0·003 | | <0·001 | |
|  | **Relationship status** | **Sole registration** | 138 | 1·34% | 23 | 0·22% | 115 | 1·12% |
|  |  | **Joint reg, separated** | 268 | 1·15% | 42 | 0·18% | 227 | 0·97% |
|  |  | **Joint registration** | 611 | 0·92% | 77 | 0·12% | 536 | 0·81% |
|  |  | **Married** | 695 | 0·73% | 64 | 0·07% | 632 | 0·66% |
|  |  | **(p-value)** | <0·001 | | <0·001 | | <0·001 | |
| **Scalds** | **Area deprivation** | **1^st^ (most deprived)** | 226 | 0·44% | NA | NA | NA | NA |
|  |  | **2^nd^** | 123 | 0·30% | NA | NA | NA | NA |
|  |  | **3^rd^** | 119 | 0·32% | NA | NA | NA | NA |
|  |  | **4^th^** | 78 | 0·22% | NA | NA | NA | NA |
|  |  | **5^th^ (least deprived)** | 45 | 0·15% | NA | NA | NA | NA |
|  |  | **(p-value)** | <0·001 | | NA | | NA | |
|  | **Social class** | **Unemployed** | 197 | 0·47% | NA | NA | NA | NA |
|  |  | **Manual** | 185 | 0·35% | NA | NA | NA | NA |
|  |  | **Intermediate** | 103 | 0·26% | NA | NA | NA | NA |
|  |  | **Professional** | 106 | 0·17% | NA | NA | NA | NA |
|  |  | **(p-value)** | <0·001 | | NA | | NA | |
|  | **Relationship status** | **Sole registration** | 55 | 0·54% | NA | NA | NA | NA |
|  |  | **Joint reg, separated** | 89 | 0·38% | NA | NA | NA | NA |
|  |  | **Joint registration** | 211 | 0·32% | NA | NA | NA | NA |
|  |  | **Married** | 236 | 0·25% | NA | NA | NA | NA |
|  |  | **(p-value)** | <0·001 | | NA | | NA | |
| **Poisonings** | **Area deprivation** | **1^st^ (most deprived)** | 554 | 1·08% | 23 | 0·04% | 532 | 1·04% |
|  |  | **2^nd^** | 361 | 0·87% | 25 | 0·06% | 336 | 0·81% |
|  |  | **3^rd^** | 299 | 0·80% | 25 | 0·07% | 274 | 0·73% |
|  |  | **4^th^** | 258 | 0·74% | 15 | 0·04% | 243 | 0·70% |
|  |  | **5^th^ (least deprived)** | 178 | 0·59% | 12 | 0·04% | 166 | 0·55% |
|  |  | **(p-value)** | <0·001 | | 0·392 | | <0·001 | |
|  | **Social class** | **Unemployed** | 475 | 1·13% | 26 | 0·06% | 449 | 1·07% |
|  |  | **Manual** | 492 | 0·94% | 27 | 0·05% | 466 | 0·89% |
|  |  | **Intermediate** | 285 | 0·71% | 23 | 0·06% | 262 | 0·65% |
|  |  | **Professional** | 398 | 0·66% | 24 | 0·04% | 374 | 0·62% |
|  |  | **(p-value)** | <0·001 | | 0·413 | | <0·001 | |
|  | **Relationship status** | **Sole registration** | 141 | 1·37% | 9 | 0·09% | 132 | 1·29% |
|  |  | **Joint reg, separated** | 301 | 1·29% | 16 | 0·07% | 285 | 1·22% |
|  |  | **Joint registration** | 606 | 0·91% | 38 | 0·06% | 569 | 0·86% |
|  |  | **Married** | 602 | 0·63% | 37 | 0·04% | 565 | 0·59% |
|  |  | **(p-value)** | <0·001 | | 0·061 | | <0·001 | |
| **Home location** | **Area deprivation** | **1^st^ (most deprived)** | 2,076 | 4·18% | 371 | 0·75% | 1,730 | 3·49% |
|  |  | **2^nd^** | 1,304 | 3·27% | 239 | 0·60% | 1,077 | 2·70% |
|  |  | **3^rd^** | 1,102 | 3·08% | 210 | 0·59% | 900 | 2·51% |
|  |  | **4^th^** | 960 | 2·88% | 202 | 0·61% | 762 | 2·29% |
|  |  | **5^th^ (least deprived)** | 771 | 2·71% | 176 | 0·62% | 601 | 2·11% |
|  |  | **(p-value)** | <0·001 | | 0·014 | | <0·001 | |
|  | **Social class** | **Unemployed** | 1,750 | 4·43% | 343 | 0·87% | 1,429 | 3·62% |
|  |  | **Manual** | 1,841 | 3·62% | 331 | 0·65% | 1,524 | 3·00% |
|  |  | **Intermediate** | 1,125 | 2·89% | 225 | 0·58% | 907 | 2·33% |
|  |  | **Professional** | 1,497 | 2·59% | 299 | 0·52% | 1,210 | 2·09% |
|  |  | **(p-value)** | <0·001 | | <0·001 | | <0·001 | |
|  | **Relationship status** | **Sole registration** | 489 | 4·92% | 112 | 1·13% | 387 | 3·89% |
|  |  | **Joint reg, separated** | 1,000 | 4·35% | 180 | 0·78% | 829 | 3·61% |
|  |  | **Joint registration** | 2,317 | 3·58% | 458 | 0·71% | 1,879 | 2·91% |
|  |  | **Married** | 2,407 | 2·69% | 448 | 0·50% | 1,975 | 2·21% |
|  |  | **(p-value)** | <0·001 | | <0·001 | | <0·001 | |
| **Non-home location** | **Area deprivation** | **1^st^ (most deprived)** | 1,089 | 2·20% | 99 | 0·20% | 992 | 2·00% |
|  |  | **2^nd^** | 840 | 2·11% | 91 | 0·23% | 752 | 1·89% |
|  |  | **3^rd^** | 701 | 1·96% | 61 | 0·17% | 642 | 1·79% |
|  |  | **4^th^** | 656 | 1·97% | 62 | 0·19% | 595 | 1·79% |
|  |  | **5^th^ (least deprived)** | 448 | 1·57% | 55 | 0·19% | 394 | 1·38% |
|  |  | **(p-value)** | <0·001 | | 0·471 | | <0·001 | |
|  | **Social class** | **Unemployed** | 917 | 2·32% | 115 | 0·29% | 806 | 2·04% |
|  |  | **Manual** | 1,057 | 2·08% | 100 | 0·20% | 959 | 1·89% |
|  |  | **Intermediate** | 754 | 1·94% | 61 | 0·16% | 694 | 1·78% |
|  |  | **Professional** | 1,006 | 1·74% | 92 | 0·16% | 916 | 1·58% |
|  |  | **(p-value)** | <0·001 | | <0·001 | | <0·001 | |
|  | **Relationship status** | **Sole registration** | 258 | 2·60% | 23 | 0·23% | 235 | 2·36% |
|  |  | **Joint reg, separated** | 594 | 2·58% | 72 | 0·31% | 525 | 2·28% |
|  |  | **Joint registration** | 1,322 | 2·05% | 150 | 0·23% | 1,176 | 1·82% |
|  |  | **Married** | 1,560 | 1·74% | 123 | 0·14% | 1,439 | 1·61% |
|  |  | **(p-value)** | <0·001 | | <0·001 | | <0·001 | |

Freq: frequency, prop: proportion

Cells containing “NA” have been suppressed either because they, or cells in the same analyses, have values <10, or because the authors elected not to run the analysis due to low prevalence

Appendix A2: List of injury types and ICD-10 admission codes

| **Injury type** | **ICD-10 codes** |
| --- | --- |
| **Any injury** | V00-X59  Y85-Y86 |
| **Transport-related** | V00-V99 |
| **Falls** | W00-W19 |
| **Striking, cutting, crushing** | W20-W23  W25-W29  W45  W50-52 |
| **Drowning/submersion** | W65-W74 |
| **Threats to breathing** | W75-W84 |
| **Smoke/fire** | X00-X09 |
| **Scalds** | X10-X19 |
| **Poisonings** | X40-X49 |

Appendix A3: Frequencies of covariates and exposures in full and complete-case samples

| **Variable** | **Category** | **Full dataset**  **(n=202,757)** | | **Complete case dataset**  **(n=195,184)** | |
| --- | --- | --- | --- | --- | --- |
|  |  | **Freq.** | **Prop.** | **Freq.** | **Prop.** |
| **Sex of child** | **Male** | 103,701 | 51·15% | 99,785 | 51·12% |
|  | **Female** | 99,056 | 48·85% | 95,399 | 48·88% |
| **Number of births in pregnancy** | **1** | 196,684 | 97·00% | 189,380 | 97·03% |
|  | **2** | 5,974 | 2·95% | 5,708 | 2·92% |
|  | **3+** | 99 | 0·05% | 96 | 0·05% |
| **Country of birth of mother** | **Scotland** | 154,266 | 76·08% | 148,981 | 76·33% |
|  | **Rest of UK / Ireland** | 20,760 | 10·24% | 19,730 | 10·11% |
|  | **Other countries** | 27,731 | 13·68% | 26,473 | 13·56% |
| **Age of mother at first live birth** | **<20** | 33,635 | 16·77% | 32,840 | 16·83% |
|  | **20-24** | 50,176 | 25·02% | 48,922 | 25·06% |
|  | **25-29** | 53,257 | 26·55% | 51,836 | 26·56% |
|  | **30-34** | 43,845 | 21·86% | 42,557 | 21·80% |
|  | **35+** | 19,641 | 9·79% | 19,029 | 9·75% |
|  | **(missing within dataset)** | 2,203 | 1·09% | NA | NA |
| **Older siblings** | **0** | 87,838 | 44·68% | 87,439 | 44·80% |
|  | **1** | 67,878 | 34·52% | 67,452 | 34·56% |
|  | **2** | 26,763 | 13·61% | 26,468 | 13·56% |
|  | **3+** | 14,134 | 7·19% | 13,825 | 7·08% |
|  | **(child missing from dataset)** | 4,274 | 2·11% | NA | NA |
|  | **(missing within dataset)** | 1,870 | 0·92% | NA | NA |
| **Area deprivation** | **1^st^ (most deprived)** | 52,862 | 26·11% | 51,176 | 26·22% |
|  | **2^nd^** | 42,655 | 21·07% | 41,299 | 21·16% |
|  | **3^rd^** | 38,967 | 19·25% | 37,496 | 19·21% |
|  | **4^th^** | 36,184 | 17·87% | 34,920 | 17·89% |
|  | **5^th^ (least deprived)** | 31,791 | 15·70% | 30,293 | 15·52% |
|  | **(missing within dataset)** | 298 | 0·15% | NA | NA |
| **Social class** | **Unemployed** | 43,780 | 21·59% | 41,949 | 21·49% |
|  | **Manual** | 54,104 | 26·68% | 52,398 | 26·85% |
|  | **Intermediate** | 41,519 | 20·48% | 40,113 | 20·55% |
|  | **Professional** | 63,354 | 31·25% | 60,724 | 31·11% |
| **Relationship status** | **Sole registration** | 10,666 | 5·26% | 10,269 | 5·26% |
|  | **Joint reg, separated** | 23,992 | 11·83% | 23,306 | 11·94% |
|  | **Joint registration** | 68,651 | 33·86% | 66,394 | 34·02% |
|  | **Married** | 99,448 | 49·05% | 95,215 | 48·78% |
| **Health board** | **Ayrshire & Arran** | 13,240 | 6·54% | 12,898 | 6·61% |
|  | **Borders** | 3,603 | 1·78% | 3,525 | 1·81% |
|  | **Dumfries & Galloway** | 4,917 | 2·43% | 4,836 | 2·48% |
|  | **Northern & Western Isles** | 2,448 | 1·21% | 2,336 | 1·20% |
|  | **Fife** | 14,361 | 7·09% | 14,106 | 7·23% |
|  | **Forth Valley** | 11,276 | 5·57% | 11,043 | 5·66% |
|  | **Grampian** | 22,020 | 10·88% | 20,517 | 10·51% |
|  | **Greater Glasgow & Clyde** | 45,586 | 22·52% | 43,882 | 22·48% |
|  | **Highland** | 10,849 | 5·36% | 10,533 | 5·40% |
|  | **Lanarkshire** | 25,485 | 12·59% | 24,727 | 12·67% |
|  | **Lothian** | 33,887 | 16·74% | 32,187 | 16·49% |
|  | **Tayside** | 14,787 | 7·30% | 14,594 | 7·48% |
|  | **(child missing from dataset)** | 298 | 0·15% | NA | NA |

Freq: frequency, prop: proportion

Cells containing “NA” refer to missing categories that did not exist in the complete-case dataset

Appendix A4: Slope (SII) indices of inequality and 95% confidence intervals for the difference between least and most advantaged children according to each socio-economic circumstance (SEC) indicator for any (vs. none), multiple (vs. 0-1), severe (vs. none/non-severe) injury, types of injury, and geographical injury location before and after adjustment

| **Injury outcome** | | **SEC** | **Unadjusted** | | **Adjusted for covariates*** | | **Adjusted for covariates* and relevant SECs**** | |
| --- | --- | --- | --- | --- | --- | --- | --- | --- |
|  |  |  | **SII** | **95% C.I.** | **SII** | **95% C.I.** | **SII** | **95% C.I.** |
| **Any injury** | **Birth to age 5** | **Area deprivation** | 2·29% | 1·96% to 2·63% | 1·06% | 0·69% to 1·42% | 0·66% | 0·29% to 1·04% |
|  |  | **Social class** | 2·73% | 2·38% to 3·08% | 1·31% | 0·89% to 1·72% | 0·97% | 0·55% to 1·40% |
|  |  | **Relationship status** | 3·37% | 3·01% to 3·74% | 1·63% | 1·21% to 2·04% | 1·39% | 0·97% to 1·82% |
|  | **Infancy only** | **Area deprivation** | 0·14% | 0·00% to 0·27% | -0·09% | -0·24% to 0·06% | -0·21% | -0·36% to -0·05% |
|  |  | **Social class** | 0·47% | 0·33% to 0·61% | 0·23% | 0·07% to 0·40% | 0·13% | -0·04% to 0·30% |
|  |  | **Relationship status** | 0·68% | 0·53% to 0·83% | 0·45% | 0·29% to 0·62% | 0·42% | 0·25% to 0·60% |
|  | **Age 1 to 5 only** | **Area deprivation** | 2·23% | 1·92% to 2·55% | 1·19% | 0·85% to 1·53% | 0·90% | 0·55% to 1·25% |
|  |  | **Social class** | 2·33% | 2·00% to 2·65% | 1·10% | 0·72% to 1·48% | 0·85% | 0·45% to 1·25% |
|  |  | **Relationship status** | 2·81% | 2·47% to 3·15% | 1·25% | 0·87% to 1·64% | 1·04% | 0·65% to 1·44% |
| **Multiple injuries***** | **Birth to age 5** | **Area deprivation** | 0·23% | 0·15% to 0·32% | 0·10% | 0·01% to 0·19% | 0·06% | -0·03% to 0·16% |
|  |  | **Social class** | 0·31% | 0·22% to 0·40% | 0·16% | 0·06% to 0·26% | 0·13% | 0·03% to 0·24% |
|  |  | **Relationship status** | 0·34% | 0·24% to 0·43% | 0·15% | 0·05% to 0·25% | 0·12% | 0·01% to 0·22% |
| **Severe injury***** | **Birth to age 5** | **Area deprivation** | 0·27% | 0·19% to 0·35% | 0·17% | 0·09% to 0·26% | 0·16% | 0·08% to 0·25% |
|  |  | **Social class** | 0·20% | 0·12% to 0·28% | 0·04% | -0·05% to 0·14% | 0·02% | -0·08% to 0·12% |
|  |  | **Relationship status** | 0·24% | 0·15% to 0·32% | 0·10% | 0·00% to 0·20% | 0·09% | 0·00% to 0·19% |
| **Falls** | **Birth to age 5** | **Area deprivation** | 0·61% | 0·38% to 0·83% | 0·26% | 0·02% to 0·51% | 0·16% | -0·09% to 0·42% |
|  |  | **Social class** | 0·57% | 0·33% to 0·80% | 0·21% | -0·07% to 0·49% | 0·06% | -0·23% to 0·35% |
|  |  | **Relationship status** | 1·09% | 0·85% to 1·34% | 0·51% | 0·23% to 0·79% | 0·45% | 0·16% to 0·75% |
|  | **Infancy only** | **Area deprivation** | -0·10% | -0·20% to 0·00% | -0·18% | -0·29% to -0·07% | -0·25% | -0·37% to -0·13% |
|  |  | **Social class** | 0·15% | 0·04% to 0·25% | 0·11% | -0·01% to 0·24% | 0·11% | -0·02% to 0·24% |
|  |  | **Relationship status** | 0·26% | 0·15% to 0·37% | 0·21% | 0·09% to 0·34% | 0·25% | 0·11% to 0·38% |
|  | **Age 1 to 5 only** | **Area deprivation** | 0·73% | 0·52% to 0·94% | 0·45% | 0·23% to 0·68% | 0·42% | 0·19% to 0·65% |
|  |  | **Social class** | 0·43% | 0·22% to 0·65% | 0·10% | -0·16% to 0·35% | -0·06% | -0·33% to 0·21% |
|  |  | **Relationship status** | 0·87% | 0·65% to 1·09% | 0·33% | 0·07% to 0·58% | 0·23% | -0·03% to 0·50% |
| **Striking, cutting, crushing injuries** | **Birth to age 5** | **Area deprivation** | 0·45% | 0·30% to 0·59% | 0·21% | 0·05% to 0·36% | 0·12% | -0·05% to 0·28% |
|  |  | **Social class** | 0·59% | 0·44% to 0·75% | 0·30% | 0·13% to 0·48% | 0·21% | 0·03% to 0·40% |
|  |  | **Relationship status** | 0·63% | 0·47% to 0·79% | 0·36% | 0·18% to 0·53% | 0·28% | 0·09% to 0·46% |
|  | **Infancy only** | **Area deprivation** | 0·07% | 0·02% to 0·12% | 0·02% | -0·04% to 0·07% | 0·00% | -0·06% to 0·06% |
|  |  | **Social class** | 0·10% | 0·04% to 0·15% | 0·03% | -0·03% to 0·10% | 0·01% | -0·05% to 0·08% |
|  |  | **Relationship status** | 0·17% | 0·11% to 0·23% | 0·09% | 0·03% to 0·16% | 0·09% | 0·03% to 0·16% |
|  | **Age 1 to 5 only** | **Area deprivation** | 0·38% | 0·24% to 0·51% | 0·19% | 0·04% to 0·33% | 0·12% | -0·04% to 0·27% |
|  |  | **Social class** | 0·50% | 0·36% to 0·64% | 0·27% | 0·10% to 0·43% | 0·20% | 0·03% to 0·37% |
|  |  | **Relationship status** | 0·47% | 0·32% to 0·62% | 0·26% | 0·09% to 0·43% | 0·18% | 0·01% to 0·36% |
| **Scalds***** | **Birth to age 5** | **Area deprivation** | 0·34% | 0·25% to 0·43% | 0·23% | 0·14% to 0·32% | 0·19% | 0·09% to 0·28% |
|  |  | **Social class** | 0·41% | 0·31% to 0·50% | 0·24% | 0·13% to 0·34% | 0·18% | 0·08% to 0·29% |
|  |  | **Relationship status** | 0·24% | 0·15% to 0·33% | 0·16% | 0·05% to 0·26% | 0·07% | -0·04% to 0·18% |
| **Poisonings** | **Birth to age 5** | **Area deprivation** | 0·61% | 0·46% to 0·75% | 0·30% | 0·15% to 0·46% | 0·22% | 0·06% to 0·37% |
|  |  | **Social class** | 0·66% | 0·51% to 0·81% | 0·32% | 0·14% to 0·49% | 0·20% | 0·02% to 0·38% |
|  |  | **Relationship status** | 0·90% | 0·74% to 1·06% | 0·39% | 0·22% to 0·57% | 0·29% | 0·11% to 0·47% |
|  | **Infancy only** | **Area deprivation** | 0·01% | -0·03% to 0·04% | -0·02% | -0·06% to 0·02% | -0·02% | -0·06% to 0·02% |
|  |  | **Social class** | 0·03% | -0·01% to 0·06% | 0·00% | -0·04% to 0·04% | 0·00% | -0·05% to 0·04% |
|  |  | **Relationship status** | 0·05% | 0·01% to 0·09% | 0·02% | -0·02% to 0·07% | 0·03% | -0·02% to 0·07% |
|  | **Age 1 to 5 only** | **Area deprivation** | 0·60% | 0·46% to 0·75% | 0·32% | 0·17% to 0·47% | 0·24% | 0·08% to 0·39% |
|  |  | **Social class** | 0·63% | 0·49% to 0·78% | 0·32% | 0·15% to 0·49% | 0·20% | 0·02% to 0·38% |
|  |  | **Relationship status** | 0·85% | 0·69% to 1·00% | 0·37% | 0·20% to 0·54% | 0·26% | 0·09% to 0·44% |
| **Home location** | **Birth to age 5** | **Area deprivation** | 1·87% | 1·59% to 2·15% | 0·85% | 0·55% to 1·14% | 0·53% | 0·22% to 0·84% |
|  |  | **Social class** | 2·36% | 2·07% to 2·65% | 1·13% | 0·79% to 1·46% | 0·78% | 0·43% to 1·14% |
|  |  | **Relationship status** | 2·61% | 2·31% to 2·91% | 1·26% | 0·93% to 1·60% | 0·94% | 0·59% to 1·30% |
|  | **Infancy only** | **Area deprivation** | 0·14% | 0·02% to 0·27% | -0·03% | -0·16% to 0·11% | -0·14% | -0·28% to 0·00% |
|  |  | **Social class** | 0·41% | 0·29% to 0·54% | 0·24% | 0·09% to 0·39% | 0·17% | 0·01% to 0·33% |
|  |  | **Relationship status** | 0·57% | 0·43% to 0·71% | 0·42% | 0·27% to 0·57% | 0·42% | 0·25% to 0·58% |
|  | **Age 1 to 5 only** | **Area deprivation** | 1·77% | 1·52% to 2·03% | 0·90% | 0·63% to 1·17% | 0·69% | 0·41% to 0·97% |
|  |  | **Social class** | 1·98% | 1·72% to 2·25% | 0·91% | 0·60% to 1·21% | 0·63% | 0·31% to 0·94% |
|  |  | **Relationship status** | 2·09% | 1·82% to 2·37% | 0·87% | 0·56% to 1·17% | 0·55% | 0·23% to 0·87% |
| **Non-home location** | **Birth to age 5** | **Area deprivation** | 0·68% | 0·46% to 0·89% | 0·35% | 0·11% to 0·58% | 0·21% | -0·03% to 0·45% |
|  |  | **Social class** | 0·72% | 0·50% to 0·95% | 0·40% | 0·14% to 0·66% | 0·24% | -0·04% to 0·51% |
|  |  | **Relationship status** | 1·13% | 0·90% to 1·36% | 0·58% | 0·31% to 0·84% | 0·46% | 0·19% to 0·74% |
|  | **Infancy only** | **Area deprivation** | 0·04% | -0·03% to 0·10% | -0·05% | -0·13% to 0·02% | -0·09% | -0·17% to -0·01% |
|  |  | **Social class** | 0·17% | 0·10% to 0·24% | 0·09% | 0·00% to 0·17% | 0·08% | -0·01% to 0·17% |
|  |  | **Relationship status** | 0·22% | 0·15% to 0·30% | 0·10% | 0·02% to 0·19% | 0·11% | 0·02% to 0·19% |
|  | **Age 1 to 5 only** | **Area deprivation** | 0·65% | 0·44% to 0·85% | 0·40% | 0·18% to 0·62% | 0·30% | 0·07% to 0·53% |
|  |  | **Social class** | 0·56% | 0·35% to 0·77% | 0·31% | 0·06% to 0·57% | 0·16% | -0·10% to 0·42% |
|  |  | **Relationship status** | 0·92% | 0·70% to 1·14% | 0·47% | 0·22% to 0·72% | 0·36% | 0·09% to 0·62% |

* Adjusted for sex of child, number of births in pregnancy, country of birth of mother, age of mother at first live birth, number of older siblings

** Adjusted for other relevant SEC exposures (area deprivation was adjusted for social class and relationship status only; relationship status was adjusted for social class; social class was adjusted for relationship status)

*** Analyses for infancy and early childhood were not run due to low case numbers

Appendix A5: Crosstabulations between non-SEC variables and any injury, from birth to age five, in infancy and age one to five

| **Variable** | **Category** | **All ages** | | **Infancy** | | **Early childhood** | |
| --- | --- | --- | --- | --- | --- | --- | --- |
|  |  | **Freq.** | **Prop.** | **Freq.** | **Prop.** | **Freq.** | **Prop.** |
| **Sex of child** | **Male** | 5,473 | 5.48% | 835 | 0.84% | 4,698 | 4.71% |
|  | **Female** | 4,193 | 4.40% | 667 | 0.70% | 3,569 | 3.74% |
|  | **(p-value)** | <0.001 | | 0.001 | | <0.001 | |
| **Number of births in pregnancy** | **1** | 9,420 | 4.97% | 1,465 | 0.77% | 8,055 | 4.25% |
|  | **2+** | 246 | 4.24% | 37 | 0.64% | 212 | 3.65% |
|  | **(p-value)** | 0.011 | | 0.243 | | 0.025 | |
| **Mother’s country of birth** | **Scotland** | 7,907 | 5.31% | 1,165 | 0.78% | 6,829 | 4.58% |
|  | **Rest of UK / Ireland** | 848 | 4.30% | 182 | 0.92% | 677 | 3.43% |
|  | **Other countries** | 911 | 3.44% | 155 | 0.59% | 761 | 2.87% |
|  | **(p-value)** | <0.001 | | <0.001 | | <0.001 | |
| **Age of mother at first live birth** | **<20** | 2,312 | 7.04% | 393 | 1.20% | 1,957 | 5.96% |
|  | **20-24** | 2,776 | 5.67% | 408 | 0.83% | 2,402 | 4.91% |
|  | **25-29** | 2,247 | 4.33% | 326 | 0.63% | 1,933 | 3.73% |
|  | **30-34** | 1,629 | 3.83% | 258 | 0.61% | 1,382 | 3.25% |
|  | **35+** | 702 | 3.69% | 117 | 0.61% | 593 | 3.12% |
|  | **(p-value)** | <0.001 | | <0.001 | | <0.001 | |
| **Older siblings** | **0** | 4,085 | 4.67% | 670 | 0.77% | 3,463 | 3.96% |
|  | **1** | 3,334 | 4.94% | 506 | 0.75% | 2,857 | 4.24% |
|  | **2** | 1,402 | 5.30% | 203 | 0.77% | 1,218 | 4.60% |
|  | **3+** | 845 | 6.11% | 123 | 0.89% | 729 | 5.27% |
|  | **(p-value)** | <0.001 | | 0.398 | | <0.001 | |

Freq: frequency, prop: proportion

Appendix A6: Relative (RII) and slope (SII) indices of inequality and 95% confidence intervals for the difference between least and most advantaged children according to each SEC indicator for any injury, falls, striking injuries and poisonings from birth to age five, in infancy and age one to five before and after adjustment with additional adjustment for health board

| **Injury outcome** | | **SEC** | | **Adjusted for other SECs only** | | **Adjusted for covariates* and other SECs** | | **Adjusted for covariates*, other SECs and health board** | |
| --- | --- | --- | --- | --- | --- | --- | --- | --- | --- |
|  |  |  |  | **R/SII** | **95% C.I.** | **R/SII** | **95% C.I.** | **R/SII** | **95% C.I.** |
| **Any injury** | **Birth to age 5** | **Area** | **RII** | 1·22 | 1·13 to 1·32 | 1·15 | 1·06 to 1·24 | 1·17 | 1·08 to 1·26 |
|  |  | **Social class** |  | 1·36 | 1·25 to 1·47 | 1·19 | 1·09 to 1·30 | 1·17 | 1·07 to 1·28 |
|  |  | **Relationship** |  | 1·63 | 1·50 to 1·76 | 1·29 | 1·18 to 1·41 | 1·29 | 1·18 to 1·41 |
|  | **Infancy only** | **Area** |  | 0·80 | 0·66 to 0·97 | 0·77 | 0·63 to 0·93 | 1·01 | 0·82 to 1·25 |
|  |  | **Social class** |  | 1·49 | 1·22 to 1·83 | 1·25 | 0·99 to 1·57 | 1·18 | 0·94 to 1·48 |
|  |  | **Relationship** |  | 2·24 | 1·82 to 2·76 | 1·84 | 1·46 to 2·32 | 1·88 | 1·49 to 2·36 |
|  | **Age 1 to 5 only** | **Area** |  | 1·33 | 1·23 to 1·44 | 1·24 | 1·14 to 1·35 | 1·21 | 1·11 to 1·32 |
|  |  | **Social class** |  | 1·33 | 1·22 to 1·45 | 1·18 | 1·07 to 1·30 | 1·17 | 1·06 to 1·29 |
|  |  | **Relationship** |  | 1·56 | 1·43 to 1·70 | 1·22 | 1·11 to 1·35 | 1·22 | 1·11 to 1·35 |
| **Falls** | **Birth to age 5** | **Area** |  | 1·12 | 1·00 to 1·26 | 1·08 | 0·96 to 1·21 | 1·11 | 0·98 to 1·25 |
|  |  | **Social class** |  | 1·06 | 0·94 to 1·19 | 1·03 | 0·90 to 1·17 | 1·02 | 0·89 to 1·16 |
|  |  | **Relationship** |  | 1·53 | 1·36 to 1·73 | 1·23 | 1·07 to 1·40 | 1·24 | 1·09 to 1·42 |
|  | **Infancy only** | **Area** |  | 0·56 | 0·43 to 0·73 | 0·56 | 0·43 to 0·73 | 0·75 | 0·57 to 1·00 |
|  |  | **Social class** |  | 1·35 | 1·03 to 1·78 | 1·30 | 0·96 to 1·77 | 1·22 | 0·90 to 1·65 |
|  |  | **Relationship** |  | 2·03 | 1·53 to 2·69 | 1·79 | 1·32 to 2·45 | 1·86 | 1·37 to 2·54 |
|  | **Age 1 to 5 only** | **Area** |  | 1·33 | 1·17 to 1·51 | 1·26 | 1·11 to 1·44 | 1·22 | 1·07 to 1·40 |
|  |  | **Social class** |  | 1·00 | 0·88 to 1·14 | 0·97 | 0·83 to 1·12 | 0·97 | 0·84 to 1·13 |
|  |  | **Relationship** |  | 1·46 | 1·27 to 1·67 | 1·14 | 0·98 to 1·32 | 1·14 | 0·99 to 1·33 |
| **Striking, cutting, crushing injuries** | **Birth to age 5** | **Area** |  | 1·23 | 1·02 to 1·47 | 1·14 | 0·95 to 1·38 | 1·36 | 1·12 to 1·66 |
|  |  | **Social class** |  | 1·54 | 1·27 to 1·87 | 1·28 | 1·03 to 1·58 | 1·23 | 0·99 to 1·52 |
|  |  | **Relationship** |  | 1·60 | 1·32 to 1·95 | 1·37 | 1·11 to 1·70 | 1·38 | 1·11 to 1·70 |
|  | **Infancy only** | **Area** |  | 1·08 | 0·64 to 1·84 | 1·00 | 0·58 to 1·72 | 1·90 | 1·06 to 3·40 |
|  |  | **Social class** |  | 1·45 | 0·83 to 2·55 | 1·13 | 0·60 to 2·12 | 1·01 | 0·54 to 1·91 |
|  |  | **Relationship** |  | 3·96 | 2·23 to 7·04 | 2·45 | 1·30 to 4·63 | 2·58 | 1·37 to 4·89 |
|  | **Age 1 to 5 only** | **Area** |  | 1·25 | 1·03 to 1·52 | 1·16 | 0·95 to 1·42 | 1·31 | 1·06 to 1·61 |
|  |  | **Social class** |  | 1·56 | 1·27 to 1·91 | 1·30 | 1·03 to 1·63 | 1·26 | 1·00 to 1·58 |
|  |  | **Relationship** |  | 1·42 | 1·15 to 1·75 | 1·27 | 1·01 to 1·59 | 1·26 | 1·01 to 1·59 |
| **Poisonings** | **Birth to age 5** | **Area** |  | 1·40 | 1·16 to 1·68 | 1·29 | 1·07 to 1·57 | 1·02 | 0·84 to 1·25 |
|  |  | **Social class** |  | 1·45 | 1·19 to 1·77 | 1·27 | 1·02 to 1·59 | 1·27 | 1·02 to 1·58 |
|  |  | **Relationship** |  | 2·19 | 1·80 to 2·67 | 1·42 | 1·14 to 1·77 | 1·45 | 1·16 to 1·81 |
|  | **Infancy only** | **Area** |  | 0·74 | 0·35 to 1·56 | 0·65 | 0·30 to 1·41 | 0·57 | 0·25 to 1·27 |
|  |  | **Social class** |  | 1·32 | 0·59 to 2·92 | 0·96 | 0·39 to 2·35 | 0·96 | 0·39 to 2·35 |
|  |  | **Relationship** |  | 2·66 | 1·18 to 6·01 | 1·78 | 0·72 to 4·39 | 1·79 | 0·72 to 4·41 |
|  | **Age 1 to 5 only** | **Area** |  | 1·46 | 1·20 to 1·77 | 1·36 | 1·11 to 1·65 | 1·06 | 0·87 to 1·31 |
|  |  | **Social class** |  | 1·46 | 1·19 to 1·79 | 1·29 | 1·03 to 1·63 | 1·29 | 1·03 to 1·62 |
|  |  | **Relationship** |  | 2·16 | 1·76 to 2·66 | 1·40 | 1·11 to 1·76 | 1·43 | 1·14 to 1·79 |
| **Injury outcome** | | **SEC** | | **Unadjusted** | | **Adjusted for covariates*** | | **Adjusted for covariates* and relevant SECs**** | |
|  |  |  |  | **R/SII** | **95% C.I.** | **R/SII** | **95% C.I.** | **R/SII** | **95% C.I.** |
| **Any injury** | **Birth to age 5** | **Area** | **SII** | 0·99% | 0·62% to 1·36% | 0·66% | 0·29% to 1·04% | 0·75% | 0·36% to 1·15% |
|  |  | **Social class** |  | 1·49% | 1·11% to 1·88% | 0·85% | 0·42% to 1·29% | 0·78% | 0·35% to 1·21% |
|  |  | **Relationship** |  | 2·40% | 2·00% to 2·80% | 1·25% | 0·82% to 1·69% | 1·27% | 0·84% to 1·71% |
|  | **Infancy only** | **Area** |  | -0·17% | -0·32% to -0·02% | -0·21% | -0·36% to -0·05% | 0·01% | -0·15% to 0·17% |
|  |  | **Social class** |  | 0·30% | 0·15% to 0·46% | 0·17% | 0·00% to 0·34% | 0·12% | -0·05% to 0·30% |
|  |  | **Relationship** |  | 0·62% | 0·46% to 0·79% | 0·47% | 0·29% to 0·64% | 0·48% | 0·30% to 0·66% |
|  | **Age 1 to 5 only** | **Area** |  | 1·20% | 0·85% to 1·54% | 0·90% | 0·55% to 1·25% | 0·79% | 0·43% to 1·16% |
|  |  | **Social class** |  | 1·21% | 0·85% to 1·56% | 0·69% | 0·29% to 1·09% | 0·66% | 0·26% to 1·06% |
|  |  | **Relationship** |  | 1·87% | 1·50% to 2·24% | 0·85% | 0·45% to 1·26% | 0·86% | 0·46% to 1·27% |
| **Falls** | **Birth to age 5** | **Area** |  | 0·25% | 0·00% to 0·50% | 0·16% | -0·09% to 0·42% | 0·23% | -0·04% to 0·50% |
|  |  | **Social class** |  | 0·13% | -0·13% to 0·39% | 0·06% | -0·23% to 0·35% | 0·04% | -0·25% to 0·33% |
|  |  | **Relationship** |  | 0·94% | 0·67% to 1·22% | 0·45% | 0·16% to 0·75% | 0·48% | 0·18% to 0·77% |
|  | **Infancy only** | **Area** |  | -0·25% | -0·37% to -0·13% | -0·25% | -0·37% to -0·13% | -0·12% | -0·24% to 0·00% |
|  |  | **Social class** |  | 0·13% | 0·01% to 0·24% | 0·11% | -0·02% to 0·24% | 0·08% | -0·05% to 0·21% |
|  |  | **Relationship** |  | 0·30% | 0·18% to 0·43% | 0·25% | 0·11% to 0·38% | 0·27% | 0·13% to 0·40% |
|  | **Age 1 to 5 only** | **Area** |  | 0·51% | 0·28% to 0·74% | 0·42% | 0·19% to 0·65% | 0·36% | 0·12% to 0·60% |
|  |  | **Social class** |  | 0·00% | -0·24% to 0·24% | -0·06% | -0·33% to 0·21% | -0·05% | -0·32% to 0·22% |
|  |  | **Relationship** |  | 0·68% | 0·43% to 0·92% | 0·23% | -0·03% to 0·50% | 0·24% | -0·02% to 0·51% |
| **Striking, cutting, crushing injuries** | **Birth to age 5** | **Area** |  | 0·18% | 0·02% to 0·34% | 0·12% | -0·05% to 0·28% | 0·27% | 0·10% to 0·44% |
|  |  | **Social class** |  | 0·38% | 0·21% to 0·54% | 0·21% | 0·03% to 0·40% | 0·18% | 0·00% to 0·36% |
|  |  | **Relationship** |  | 0·41% | 0·24% to 0·58% | 0·28% | 0·09% to 0·46% | 0·28% | 0·09% to 0·46% |
|  | **Infancy only** | **Area** |  | 0·01% | -0·05% to 0·06% | 0·00% | -0·06% to 0·06% | 0·07% | 0·00% to 0·13% |
|  |  | **Social class** |  | 0·04% | -0·02% to 0·10% | 0·01% | -0·05% to 0·08% | 0·00% | -0·06% to 0·07% |
|  |  | **Relationship** |  | 0·14% | 0·08% to 0·21% | 0·09% | 0·03% to 0·16% | 0·10% | 0·03% to 0·16% |
|  | **Age 1 to 5 only** | **Area** |  | 0·17% | 0·02% to 0·32% | 0·12% | -0·04% to 0·27% | 0·21% | 0·05% to 0·37% |
|  |  | **Social class** |  | 0·34% | 0·18% to 0·50% | 0·20% | 0·03% to 0·37% | 0·18% | 0·00% to 0·35% |
|  |  | **Relationship** |  | 0·27% | 0·11% to 0·43% | 0·18% | 0·01% to 0·36% | 0·18% | 0·01% to 0·36% |
| **Poisonings** | **Birth to age 5** | **Area** |  | 0·28% | 0·12% to 0·44% | 0·22% | 0·06% to 0·37% | 0·02% | -0·15% to 0·19% |
|  |  | **Social class** |  | 0·31% | 0·15% to 0·48% | 0·20% | 0·02% to 0·38% | 0·20% | 0·01% to 0·38% |
|  |  | **Relationship** |  | 0·66% | 0·49% to 0·82% | 0·29% | 0·11% to 0·47% | 0·31% | 0·13% to 0·49% |
|  | **Infancy only** | **Area** |  | -0·02% | -0·06% to 0·02% | -0·02% | -0·06% to 0·02% | -0·03% | -0·08% to 0·01% |
|  |  | **Social class** |  | 0·01% | -0·03% to 0·05% | 0·00% | -0·05% to 0·04% | 0·00% | -0·05% to 0·04% |
|  |  | **Relationship** |  | 0·05% | 0·01% to 0·09% | 0·03% | -0·02% to 0·07% | 0·03% | -0·02% to 0·08% |
|  | **Age 1 to 5 only** | **Area** |  | 0·30% | 0·15% to 0·45% | 0·24% | 0·08% to 0·39% | 0·05% | -0·11% to 0·21% |
|  |  | **Social class** |  | 0·30% | 0·14% to 0·46% | 0·20% | 0·02% to 0·38% | 0·20% | 0·02% to 0·38% |
|  |  | **Relationship** |  | 0·61% | 0·44% to 0·77% | 0·26% | 0·09% to 0·44% | 0·28% | 0·10% to 0·45% |

* Adjusted for sex of child, number of births in pregnancy, country of birth of mother, age of mother at first live birth, number of older siblings

Appendix A7: Relative (RII) and slope (SII) indices of inequality and 95% confidence intervals for the difference between least and most advantaged children with at least one admission for any injury, according to each SEC indicator for multiple and severe injury, from birth to age five before and after adjustment*

| **Injury outcome** | **SEC** | | **Unadjusted** | | **Adjusted for covariates*** | | **Adjusted for covariates* and relevant SECs**** | |
| --- | --- | --- | --- | --- | --- | --- | --- | --- |
|  |  |  | **R/SII** | **95% C.I.** | **R/SII** | **95% C.I.** | **R/SII** | **95% C.I.** |
| **Multiple injury vs· one injury** | **Area** | **RII** | 1·38 | 1·04 to 1·83 | 1·12 | 0·82 to 1·52 | 1·05 | 0·77 to 1·45 |
|  | **Social class** |  | 1·69 | 1·27 to 2·27 | 1·35 | 0·95 to 1·92 | 1·31 | 0·91 to 1·88 |
|  | **Relationship** |  | 1·61 | 1·20 to 2·16 | 1·22 | 0·86 to 1·73 | 1·14 | 0·80 to 1·64 |
| **Severe injury vs· non-severe** | **Area** |  | 1·77 | 1·31 to 2·40 | 1·58 | 1·14 to 2·20 | 1·62 | 1·16 to 2·28 |
|  | **Social class** |  | 1·25 | 0·92 to 1·70 | 0·92 | 0·63 to 1·33 | 0·89 | 0·61 to 1·30 |
|  | **Relationship** |  | 1·29 | 0·94 to 1·76 | 1·11 | 0·76 to 1·61 | 1·14 | 0·77 to 1·67 |
| **Multiple injury vs· one injury** | **Area** | **SII** | 1·89% | 0·25% to 3·52% | 0·68% | -1·10% to 2·45% | 0·31% | -1·51% to 2·14% |
|  | **Social class** |  | 3·05% | 1·36% to 4·75% | 1·71% | -0·32% to 3·74% | 1·53% | -0·55% to 3·61% |
|  | **Relationship** |  | 2·76% | 1·04% to 4·49% | 1·15% | -0·87% to 3·18% | 0·81% | -1·27% to 2·89% |
| **Severe injury vs· non-severe** | **Area** |  | 2·94% | 1·38% to 4·51% | 2·36% | 0·68% to 4·04% | 2·49% | 0·75% to 4·23% |
|  | **Social class** |  | 1·16% | -0·42% to 2·75% | -0·45% | -2·36% to 1·47% | -0·59% | -2·56% to 1·37% |
|  | **Relationship** |  | 1·29% | -0·33% to 2·91% | 0·52% | -1·40% to 2·44% | 0·65% | -1·31% to 2·62% |

* Adjusted for sex of child, number of births in pregnancy, country of birth of mother, age of mother at first live birth, number of older siblings

** Adjusted for other relevant SEC exposures (area deprivation was adjusted for social class and relationship status only; relationship status was adjusted for social class; social class was adjusted for relationship status)

Appendix A8: Frequency distributions for injuries at from birth to age five, infancy and age one to five, full sample

| **Injury** | **Full sample (n=202,757)** | | | | | |
| --- | --- | --- | --- | --- | --- | --- |
|  | **Birth to age 5** | | **Infancy only** | | **Age 1 to 5 only** | |
| **Any injury** | 10,010 | 4·94% | 1,551 | 0·76% | 8,565 | 4·22% |
| **Multiple (2+) injuries** | 577 | 0·28% | NA | NA | NA | NA |
| **Severe injury** | 511 | 0·25% | NA | NA | NA | NA |
| **Transport-related** | 150 | 0·07% | NA | NA | NA | NA |
| **Falls** | 4,445 | 2·19% | 860 | 0·42% | 3,613 | 1·78% |
| **Struck, cut, crush** | 1,786 | 0·88% | 209 | 0·10% | 1,581 | 0·78% |
| **Drowning/submersion** | 12 | 0·01% | NA | NA | NA | NA |
| **Threats to breathing** | 60 | 0·03% | NA | NA | NA | NA |
| **Smoke/fire** | 45 | 0·02% | NA | NA | NA | NA |
| **Scalds** | 611 | 0·30% | NA | NA | NA | NA |
| **Poisonings** | 1,713 | 0·84% | 105 | 0·05% | 1,609 | 0·79% |
| **Admission from home** | 6,552 | 3·23% | 1,270 | 0·63% | 5,340 | 2·63% |
| **Admission from non-home location** | 3,915 | 1·93% | 398 | 0·20% | 3,526 | 1·74% |

Appendix A9: RII of area-level inequalities in any injury in infancy after adjustment for specific covariates

| **Adjusted for variable** | **RII of SIMD** | **95% C.I. of SIMD** |
| --- | --- | --- |
| Unadjusted | 1·20 | 1·01 to 1·43 |
| Sex of child | 1·20 | 1·01 to 1·43 |
| Non-singleton | 1·20 | 1·00 to 1·43 |
| Mother's country of birth | 1·24 | 1·04 to 1·49 |
| Age of mother at first live birth | 0·86 | 0·71 to 1·04 |
| Older siblings | 1·19 | 1·00 to 1·42 |
| Social class of mother | 0·97 | 0·80 to 1·17 |
| Relationship status of parents | 0·88 | 0·73 to 1·07 |

Appendix A10: Risk ratios and 95% confidence intervals for any injury in infancy, according to area deprivation quintiles and deciles (in the Scottish Index of Multiple Deprivation), after adjustment*

| **SIMD scale** | | **RR** | **95% C.I.** |
| --- | --- | --- | --- |
| **Quintiles** | **1^st^ (most deprived)** | 0·77 | 0·64 to 0·91 |
|  | **2^nd^** | 0·75 | 0·63 to 0·90 |
|  | **3^rd^** | 0·77 | 0·65 to 0·92 |
|  | **4^th^** | 0·83 | 0·69 to 0·99 |
|  | **5^th^ (least deprived)** | Reference | |
| **Deciles** | **1^st^ (most deprived)** | 0·76 | 0·59 to 0·97 |
|  | **2^nd^** | 0·82 | 0·64 to 1·05 |
|  | **3^rd^** | 0·82 | 0·64 to 1·05 |
|  | **4^th^** | 0·73 | 0·56 to 0·94 |
|  | **5^th^** | 0·74 | 0·57 to 0·95 |
|  | **6^th^** | 0·86 | 0·67 to 1·12 |
|  | **7^th^** | 0·81 | 0·63 to 1·06 |
|  | **8^th^** | 0·89 | 0·69 to 1·15 |
|  | **9^th^** | 1·05 | 0·82 to 1·36 |
|  | **10^th^ (least deprived)** | Reference | |

* Adjusted for sex of child, number of births in pregnancy, country of birth of mother, age of mother at first live birth, social class and relationship status
